# On the molecular mechanisms of mitotic kinase activation

**DOI:** 10.1098/rsob.120136

**Published:** 2012-11

**Authors:** Richard Bayliss, Andrew Fry, Tamanna Haq, Sharon Yeoh

**Affiliations:** 1 Department of Biochemistry, Henry Wellcome Laboratories for Structural Biology, University of Leicester, Lancaster Road, Leicester LE1 9HN, UK; 2 Centre for Translational Therapeutics, Henry Wellcome Laboratories for Structural Biology, University of Leicester, Lancaster Road, Leicester LE1 9HN, UK

**Keywords:** mitosis, protein kinases, regulation, protein structures

## Abstract

During mitosis, human cells exhibit a peak of protein phosphorylation that alters the behaviour of a significant proportion of proteins, driving a dramatic transformation in the cell's shape, intracellular structures and biochemistry. These mitotic phosphorylation events are catalysed by several families of protein kinases, including Auroras, Cdks, Plks, Neks, Bubs, Haspin and Mps1/TTK. The catalytic activities of these kinases are activated by phosphorylation and through protein–protein interactions. In this review, we summarize the current state of knowledge of the structural basis of mitotic kinase activation mechanisms. This review aims to provide a clear and comprehensive primer on these mechanisms to a broad community of researchers, bringing together the common themes, and highlighting specific differences. Along the way, we have uncovered some features of these proteins that have previously gone unreported, and identified unexplored questions for future work. The dysregulation of mitotic kinases is associated with proliferative disorders such as cancer, and structural biology will continue to play a critical role in the development of chemical probes used to interrogate disease biology and applied to the treatment of patients.

## 2. Protein kinases in mitotic regulation

**Figure 1.**
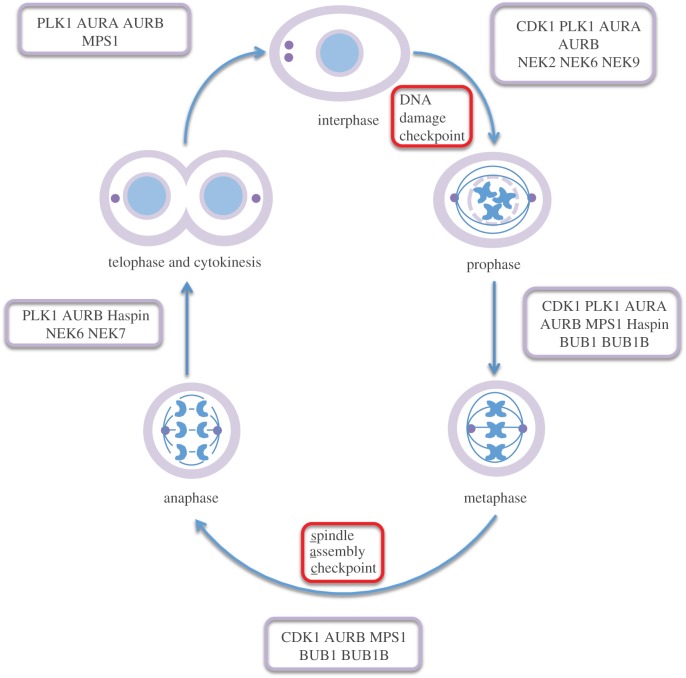
An illustration of the protein kinases that are discussed in this review and the mitotic events they coordinate. Many of the kinases play roles in multiple stages of mitosis. Progress through the cell cycle is restricted by checkpoints that serve to maintain the fidelity of genetic transmission (red boxes).

The eukaryotic cell cycle can be very broadly divided into two main stages: mitosis and interphase. During mitosis, the condensed chromosomes segregate into two genetically identical daughter cells so that each daughter cell contains a full set of chromosomes and a single centrosome. Chromosome segregation is carried out by the mitotic spindle, a dynamic molecular machine formed from microtubules and hundreds of other proteins. The fidelity of chromosome segregation is enhanced by cell-cycle checkpoints such as the spindle assembly checkpoint (SAC), which monitors the assembly of the mitotic spindle and delays chromosome segregation until every chromosome is correctly attached to spindle microtubules via kinetochores [1]. Mitotic events require precise control and coordination, involving a large number of proteins, including protein kinases, working together to choreograph the interplay between chromosomes and microtubules (figure 1). The activities of a subset of protein kinases are crucial for the proper regulation of mitosis, and disruption of any of these proteins can generate an aberrant mitotic phenotype. Dysregulation of mitotic kinases can result in severe mitotic defects that result in aneuploidy and genetic instability. These defects can subsequently give rise to a variety of diseases of chromosomal aberrations, such as cancer and Down's syndrome [2–4]. Mitotic kinases are attractive cancer drug targets as alternatives to current anti-mitotic drugs used in the clinic, which function by binding microtubules. The expectation is that compounds targeting mitotic kinases should exhibit fewer dose-limiting side effects as they would not target non-dividing cells [5]. The elaboration of the functions of mitotic kinases is a highly active subject of research and the state of play has been recently reviewed [6].

The activities of mitotic kinases are regulated in both space and time by post-translational modifications and protein–protein interactions. The most common forms of post-translational modification are phosphorylation (usually activating the kinase) and ubiquitination (generally targeting the kinase to the proteasome for degradation). Protein-binding partners can either stimulate or activate kinase activity, and can specify the subcellular localization of a population of the kinase. Unravelling these mechanisms of regulation will be crucial for understanding the aetiology of some human diseases and developing new treatments for patients. The regulatory mechanisms of these kinases have previously been compared individually with those of the archetypal examples such as protein kinase A (PKA) or cyclin-dependent kinases (Cdks). We feel that a cross-comparison of mitotic kinases will be helpful for those in the mitosis field, and to highlight the ways in which these kinases diverge from archetypal kinases.

In this review, we appraise the available structural information on the mitotic kinases and describe how this has revealed mechanisms of regulation at the molecular level. There are many protein kinases that influence mitotic events, but for reasons of brevity and clarity, this review will focus on the subset of kinase families that have members that are generally agreed to carry out their main function during mitosis, and for which there are available crystal structures: Cdk, Plk, Aurora, Nek, Bub, Haspin and Mps1/TTK. We will describe the crystal structures that have been determined, summarize the important conserved structural features that are crucial for activity and activation, and explain the key mechanisms of their regulation (table 1).

**Table 1.** Summary of key motifs found in mitotic protein kinases, with non-canonical residues emphasized in bold.

kinase	spine β4, αC, DFG HRD	DFG	HRD	activation loop phosphorylated	Lys–Glu pair	coordinate phos? (αC, β9, HRD, other)^a^	GT motif
CDK2 (*Homo sapiens*) 1HCK (autoinhibited) 1FIN (Cyclin A) 1JST (Cyclin A, pT160)	Leu66 Leu55 Phe146 His125	DFG: 145–147	HRD: 125–127	Thr160	Lys33 Glu51	Arg50 Arg150 Arg126	**Val164** Thr165
Aurora-A (*Homo sapiens*) 1MUO, 1MQ4, 1OL6, 2WTV 1OL5 (TPX2, pT288) 1OL7 (pT288)	Leu196 **Gln185** Phe275 His254	DFG: 274–276	HRD: 254–256	Thr287, Thr288	Lys162 Glu181	Arg180 **Val279** Arg255 **Arg286 (AL)**	Gly291 Thr292
Aurora B (*Xenopus laevis*) 2BFX (INCENP, pT232)	Leu140 (Met156) **Gln129** (Gln145) Phe219 (Phe235) His198 (His214)	DFG: 218–220 (DFG: 234–236)	HRD: 198–200 (HRD: 214–216)	Thr232 (Thr248)	Lys106 Glu125 (Lys122 Glu141)	Arg246 **Val239** Arg215 **Arg246 (AL)**	Gly235 Thr236
Plk1 (zebrafish) 3D5W (phospho) 3D5U (non-phospho)	Phe116 (Phe102) His105 (His91) Phe195 (Phe181) His174 (His160)	DFG: 194–196 (DFG: 180–182)	HRD: 174–176 (HRD: 160–162)	Thr210 (Thr196)	Lys82 Glu101 (Lys68 Glu87)	**Met100 (Thr86)** **Thr199 (Thr185)** Arg175 (Arg161) **Lys208 (Lys194) (AL)**	Gly213 Thr214
Nek2 (*Homo sapiens*) 2W5B, 2JAV (inactive)	Tyr70 Leu59 Phe160 His139	DFG: 159–161	HRD: 139–141	Thr170, Ser171, Thr175	Lys37 Glu55	***Ala47*** *Arg164* *Arg140*	Gly178 Thr179
Nek7 (*Homo sapiens*) 2WQM	Tyr97 Leu86 Leu180 His159	**DLG**: 179–181	HRD: 159–161	Ser195	Lys63 Glu82	*Lys81* *Arg160* *Arg184*	Gly198 Thr199
Haspin (*Homo sapiens*) 2VUW, 2WB8	Leu559 **Ser539** Tyr688 His647	**DYT**: 687–689	HRD: 647–649	not phosphorylated	Lys511 Glu535	***Pro534*** *Arg692* *Arg648*	**Tyr722**^b^
Bub1 (*Homo sapiens*) 3E7E	Phe852 **Gly834** Leu947 His915	**DLG**: 946–948	**HGD**: 915–917	not phosphorylated	Glu830 Lys821	Not phosphorylated	Gly959 Thr960 **Glu967** Thr968
Mps1 (*Homo sapiens*) 2ZMC (apo) 3H9F (phospho)	Leu588 Leu575 Phe665 His645	DFG: 664–666	**HSD**: 644–647	Thr675, Thr676, Thr686	Lys553 Glu571	***Asn570*** ***Asn669*** ***Ser646*** ***Lys681* (*AL*)** ***Lys708, Lys710* (*see text*)**	Gly685 Thr686

^a^*α*C is residue preceding Glu, β9 is residue DFG+3, AL is activation loop two residues preceding phosphorylated Ser/Thr, italics show predicted residues where no relevant structure exists.

^b^Functionally equivalent to GT motif in that the side chain forms H-bond with catalytic Asp649.

## 3. Families of mitotic kinases

There are 518 known protein kinases in the human kinome that are divided by primary sequence into seven groups [7]. With the exception of Cdks and Lats kinases, which belong in the CMGC and AGC families, respectively, most mitotic kinases are somewhat divergent from archetypal members of the seven groups. As we will see, this divergence is also reflected in their structures and specific regulatory mechanisms. We briefly outline the functions and regulation of selected mitotic kinase families below, and refer to the many excellent reviews that are available for further details.

### 3.1. Cyclin-dependent kinases

Cdks are a large family of serine/threonine kinases that have been well studied since their discovery in the 1970s [8]. Cdks become active when bound to their cognate cyclin partner protein, and subsequently by phosphorylation by a Cdk-activating kinase (CAK) [9,10]. As their name suggests, the level of cyclin protein expression, and hence Cdk activity, varies through the cell cycle. Hence, different Cdk/cyclin complexes function in distinct phases of the cell cycle, with, for example, Cdk2/Cyclin E associated with G1/S progression, and Cdk1/Cyclin B associated with M-phase entry.

Cdk1 was first discovered in *Saccharomyces cerevisiae* (cdc28) and in *Schizosaccharomyces pombe* (cdc2), and is the key regulator of the G2/M transition, when it is subject to control by the DNA damage checkpoint [11]. Cdk1 is regulated by inhibitory phosphorylation on Thr14 and Tyr15, which is reversed by phosphatases of the Cdc25 family [9]. Cdk1 forms a complex with Cyclin B, and is then phosphorylated by CAK. Cdk1/Cyclin B activity drives cells into mitosis through phosphorylation of many substrates that contribute to chromosome condensation, nuclear envelope breakdown and spindle assembly [11]. Cdk1/Cyclin B also stimulates activation of the anaphase-promoting complex/cyclosome (APC/C), which is required to trigger chromosome segregation through proteolytic degradation of securin, and mitotic exit through degradation of cyclin B, thereby leading to the loss of cellular Cdk1 activity.

### 3.2. Polo-like kinases

There are four Polo-like kinases (Plk1–4), all of which are proposed to have functional roles within the cell cycle [12,13]. Plks are serine/threonine kinases, which possess an N-terminal catalytic domain, a destruction box (D-box) and a C-terminal region containing either one or two polo-box domains [14]. The best-characterized member of the family is Polo-like-kinase-1 (Plk1), which is known to play an essential role in many processes associated with cell division, including mitotic entry, centrosome separation, bipolar spindle assembly and cytokinesis. Expression of Plk1 is highest in late G2, where it regulates Cdk1/Cyclin B activity through phosphorylation of the phosphatase Cdc25, and in M phase, where it has many substrates. Plk1 is activated through phosphorylation on its activation loop by upstream kinases including Aurora-A, and phosphorylation of Plk1 peaks between metaphase and anaphase [15]. In late mitosis, Plk1 is ubiquitinated by the APC/C, leading to its degradation by the proteasome [12,16].

### 3.3. Aurora kinases

The first members of the Aurora family of serine/threonine protein kinases were discovered in yeast and *Drosophila* [13,17,18]. Although yeast have only a single Aurora kinase, and *Drosophila* have two, there are three members of this family in humans: Aurora-A, -B and -C. Aurora-A is associated with centrosomes and the mitotic spindle, and functions in mitotic entry, centrosome maturation and mitotic spindle assembly, and phosphorylates microtubule-associated proteins such as TACC3 [19,20]. Aurora-A also has non-mitotic roles in neurite extension and in stimulating the disassembly of cilia [21,22]. During early mitosis, Aurora-B associates with chromosomes, and phosphorylates histone H3 on Ser10 and Ser28 [23]. Aurora-B is part of the chromosome passenger complex (CPC), a key component of the SAC and also functions in cytokinesis [24]. Aurora-C is expressed only in a few tissues and is believed to fulfil a similar function to Aurora-B [25].

All three Aurora kinases are activated by phosphorylation and through the binding of partner proteins, and are targeted for degradation by the APC/C. They have two domains: an N-terminal domain that includes regulatory elements and a C-terminal catalytic domain. The regulatory domain varies greatly among the family, whereas the C-terminal catalytic domains are about 70 per cent homologous [26].

### 3.4. Never in mitosis A-related kinases

Never in mitosis A (NIMA)-related serine/threonine kinases (Neks) were first identified in *Aspergillus nidulans,* where NIMA was shown to be essential for progression through the cell cycle [27,28]. There are 11 members of the Nek family in humans, of which Neks 2, 6, 7 and 9 are strongly implicated in mitotic function [29]. Of the human Nek kinases, all but Nek6 and Nek7 have a C-terminal region that encodes coiled-coil motifs and/or β-propeller domains of the regulator of chromosome condensation 1 family. Nek2 and Nek9 are believed to form homodimers through their coiled-coil motifs [30,31].

Nek2 has the highest homology at an amino acid level to the archetypal NIMA protein from *Aspergillus* and functions in centrosome separation by phosphorylating tethering proteins such as C-Nap1 [32,33]. Nek2 activity peaks during the transition from G2 to M phase after protein phosphatase 1 (PP1) inactivation and phosphorylation [34]. Once cells enter mitosis, Nek2 is ubiquitinated by APC/C-Cdc20 and then degraded [35,36]. Nek9 is upstream of Neks 6 and 7 in a recently characterized mitotic pathway that functions in bipolar spindle assembly and centrosome separation [29,37]. Nek9 is thought to activate Nek6 and Nek7 through phosphorylation and allosteric mechanisms [38,39]. Very few downstream substrates of these kinases have been identified, with the exception of the microtubule motor protein Eg5, which is a Nek6 substrate in centrosome separation, and Nedd1, which is a Nek9 substrate in the recruitment of γ-tubulin to centrosomes [40,41].

### 3.5. Budding uninhibited by benzimidazoles

Budding uninhabited by benzimidazole (Bub) proteins were first discovered in *S. cerevisiae*, and there are two known members of this family of serine/threonine protein kinases in humans: Bub1 and Bub1B/BubR1. There are several recent reviews that describe their functions, which we briefly describe below [42–44].

Bub1 and BubR1 both localize to kinetochores and are thought to play dual roles, contributing to the SAC and to the regulation of kinetochore–MT attachments. The precise contribution of the catalytic activities of Bubs to the SAC is under active investigation, and many substrates of these kinases are yet to be discovered. These proteins also have scaffolding roles. Bub1 localizes to kinetochores in early prophase, and determines kinetochore recruitment of a number of proteins, including BubR1 and an adaptor protein Bub3. The kinetochore localization of Bub1 and BubR1 depend on their N-terminal TPR domains. Both kinases interact with the β-propeller domain of Bub3 through their Gle2-binding sequence motifs. These interactions have been characterized by X-ray crystallography [45–47].

### 3.6. Haploid germ-cell-specific nuclear protein kinase

Haploid germ-cell-specific nuclear protein kinase (Haspin) is a serine/threonine protein kinase that has an atypical catalytic domain that is highly divergent from other eukaryotic protein kinases at the sequence level [48]. Haspin was first identified in mice as the product of the germ-cell-specific gene-2 and is conserved in other eukaryotes, including *S. cerevisiae*. Haspin depletion results in defective chromosome alignment on the metaphase plate and a delay in mitotic exit due to activation of the SAC, thought to arise in part from loss of chromosome cohesion. The most clearly defined substrate of Haspin is residue Thr3 of histone H3 (HH3-Thr3), a mitosis-specific modification that is required to localize the CPC to centromeres and activate Aurora-B [49,50]. The domain structure of Haspin comprises a C-terminal catalytic domain and an N-terminal region of unknown structure that is highly divergent between Haspin homologues. Haspin protein levels and catalytic activity appear to be constant through the cell cycle. Indeed, although Aurora-B phosphorylates Haspin, leading to an increase in the phosphorylation of HH3-Thr3, this is not through an increase in the intrinsic activity of Haspin [51]. Therefore, regulation of the phosphorylation of Haspin substrates such as HH3-Thr3 is thought to occur through alternative mechanisms such as phosphatases, or through the binding of a protein that inhibits Haspin activity.

### 3.7. Monopolar spindle-1

Monopolar spindle-1 (Mps1) is a dual-specificity kinase that was first identified in *S. cerevisiae* as having multiple cell-cycle functions, including duplication of the spindle pole body, the functional equivalent of the centrosome and the SAC (reviewed by Liu & Winey [52]). Human Mps1, also known as TTK, is required for proper chromosome alignment on the metaphase plate and for the fidelity of chromosome segregation, and might also have a role at centrosomes. Evidence is clearest for a role in the SAC, as depletion of Mps1 by RNAi or blocking Mps1 activity using small molecule inhibitors abrogates the checkpoint and markedly reduces the duration of mitosis (reviewed by Lan & Cleveland [53]). One key contribution of Mps1 to the SAC is in recruitment of other checkpoint components to the kinetochore, including Mad1, Mad2, Bub1, BubR1 and Bub3.

The catalytic domain of Mps1 is in the C-terminal region, and the N-terminal region includes TPR repeats that promote homodimer formation, which is important for proper Mps1 function [54]. Mps1 becomes phosphorylated on multiple sites during mitosis, which is at least partly due to autophosphorylation, and phospho-Mps1 is localized to both kinetochores and centrosomes [55]. In late mitosis, Mps1 is ubiquitinated by the APC/C and subsequently degraded [56].

## 4. The hallmarks of active kinase architecture

**Figure 2.**
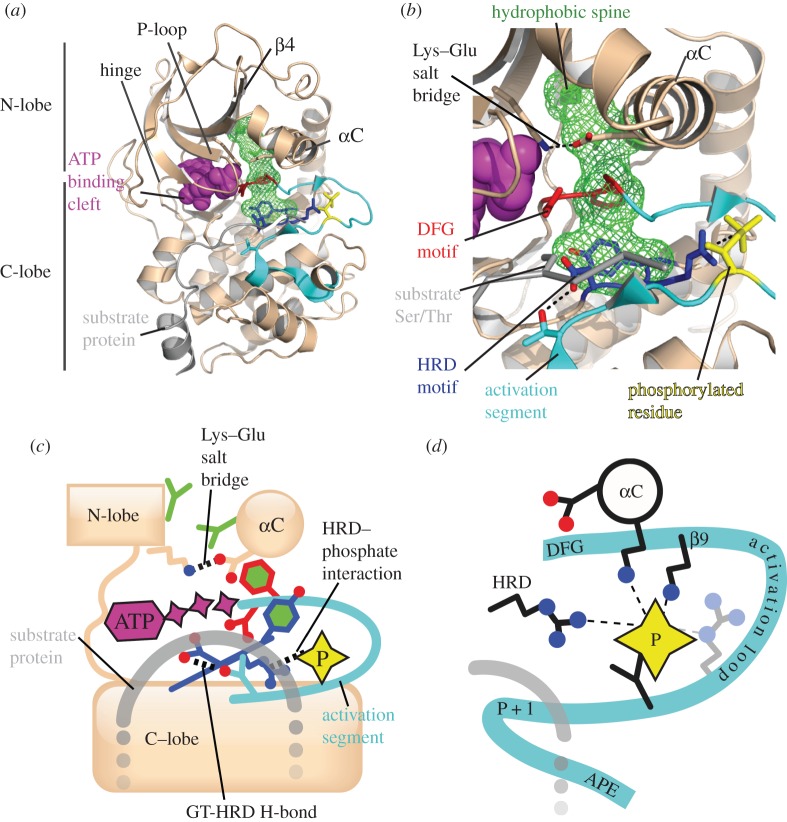
Conserved structural features of the active conformation of a protein kinase. The four panels use the following colour scheme: kinase main chain and Lys/Glu pair (beige), HRD motif (blue), DFG motif side chains (red), activation segment (cyan), phosphorylated residue on activation loop (yellow), hydrophobic spine (green), ATP (magenta), substrate protein (grey). (*a*) Architecture of the archetypal protein kinase A (PKA) catalytic domain in cartoon representation, with key residues shown as sticks and the ADP ligand shown as spheres (PDB code 1JBP). The side chains of the four residues that comprise the hydrophobic spine are surrounded by a wire mesh. This and all subsequent structure figures were produced using PyMOL. (*b*) Magnified view of the active site and residues involved in regulation. Key interactions between residues are indicated with dashed black lines; these do not necessarily imply hydrogen bonds. (*c*) Schematic that summarizes the major features of the kinase active conformation. Versions of this diagram will be used throughout the text to illustrate differences between inactive and active states of protein kinases. Three key interactions are labelled in black. (*d*) Schematic diagram to illustrate the canonical interactions between the phosphorylated residue of the activation loop and basic residues in the αC-helix, β9 and the HRD motif. These interactions are not all conserved in mitotic kinases. For example, Aurora-A and Plk1 lack the basic residue on β9, and instead have a basic residue on the activation loop (shown with fainter colouring). The different parts of the activation segment are labelled, such as the P+1 loop that recognizes the P+1 residue of the substrate protein.

The first crystal structure of a protein kinase was of PKA in its active form, determined by the Taylor laboratory [57]. This structure serves as a model for understanding the features of an active kinase. Structures of well over a hundred protein kinases have been determined, and these have yielded valuable insights into their mechanisms of catalysis and regulation [58–60]. The protein kinase fold is composed of two sub-domains: the N-terminal lobe (N-lobe) and the C-terminal lobe (C-lobe), connected by a flexible hinge region (figure 2*a*). The N-lobe is made up of a five-stranded β-sheet and (usually) two α-helices, of which the αC-helix is the structurally most conserved, whereas the larger C-lobe is mainly α-helical. A molecule of ATP binds in the cleft between the two lobes. The adenosine base forms H-bonds with the kinase hinge region, the ribose moiety binds to the ribose binding pocket and the phosphate groups interact with the Gly-rich loop, which is also called the phosphate-binding loop (P-loop). Sites phosphorylated by kinases usually lie within unstructured regions of proteins, and structural studies using substrate peptides have shown that the C-lobe recognizes the sequence on either side of the phosphorylated residues [61,62]. In this review, we will not go into any further depth regarding the catalytic mechanism of kinases or how they recognize ATP and protein substrates, which have been reviewed elsewhere [59]. Instead, we will focus on the key features of protein kinase structure, and explain how structural changes are related to different states of activity among mitotic kinases. A set of highly conserved motifs form the catalytic core and key regulatory features of protein kinases, as detailed below (figure 2*b*,*c*). Intriguingly, most mitotic kinases are divergent in at least one of these canonical motifs, perhaps reflecting their unusual regulatory mechanisms (table 1).

The *HRD and DFG motifs* are defining features of protein kinases, and the aspartic acids in these motifs are essential catalytic residues. The aspartate residue of the HRD motif acts as a base catalyst that deprotonates the substrate side chain serine/threonine, and this region of the kinase is sometimes referred to as the catalytic loop [63]. The aspartate of the DFG motif coordinates a magnesium ion that activates the gamma phosphate of ATP, and this region of kinases is often called the magnesium (Mg^2+^)-binding loop. As with any enzyme, these residues must be positioned correctly and in the appropriate chemical environment in order for the kinase to catalyse phosphate transfer. In active kinases, the DFG motif adopts the DFG-in conformation, and contacts the HRD motif through a hydrophobic interaction. Displacement of these motifs into positions incompatible with catalysis is part of the regulatory mechanism of many kinases and can also be induced by small molecule inhibitors of kinases, as exemplified by the DFG-out conformation of cAbl that is induced by Imatinib/Glivec, used in the treatment of chronic myelogenous leukaemia and gastrointestinal stromal tumours [64].

The *lysine–glutamate salt bridge* between the ammonium group of the lysine and the carboxylate group of the glutamate is a characteristic feature of active protein kinases. The lysine is located on the β3-strand and the glutamate is located on the αC-helix, and the bridge thus links these two important structural elements of the N-lobe together. The lysine also interacts with the terminal phosphate groups of ATP, stabilizing them in the correct position for catalysis [59].

The *hydrophobic spine* is a recently defined feature of active kinase structures and is made up of the side chains of four hydrophobic residues (figure 2 and table 1). The first side chain comes from the β4 strand in the N-terminal lobe; the second comes from the adjacent αC helix; the third is the phenylalanine residue from the DFG motif; and, finally, the fourth member of the spine is the histidine residue from the HRD motif [65]. The relative positions of these four residues are strikingly similar in active kinase structures, where they form a continuous hydrophobic chain, or ‘spine’, shown as a green wire mesh in figure 2*b*.

The *activation segment* starts at the DFG motif and ends at a sequence that has the consensus alanine–proline–glutamic acid, called the APE motif (figure 2*d*). It is often called the T-loop or activation loop, but we will reserve this latter term for a portion of the activation segment that contains the primary site of activating phosphorylation, following the same naming convention as Nolen *et al.* [60]. The activation segment includes several important features that are conserved in active kinase structures. Strand β9 pairs with β6 within the catalytic loop, immediately N-terminal to the HRD motif. The activation loop is variable in length and sequence, and is the primary site of regulatory phosphorylation [59]. The P + 1 loop forms a pocket that interacts with the P + 1 site of substrates, and must therefore form the right conformation for efficient catalysis. In serine/threonine kinases, this requires a conserved H-bond between a threonine (or serine) within the P + 1 loop, which is usually preceded by a glycine (forming a conserved GT motif), and the aspartic acid of the HRD motif. In tyrosine kinases, the residue equivalent to the threonine of the GT motif is usually a proline.

The *phosphorylated residue on the activation loop* is typically coordinated by the side chains of basic residues at specific positions on three different structural features, namely the residue preceding the glutamic acid on αC, the residue three positions C-terminal to the DFG motif on β9 and the arginine residue of the HRD motif (figure 2*d*). This three-point attachment helps to stabilize the activation segment in the correct conformation for substrate binding.

Every mitotic kinase that has been crystallized diverges from the canonical sequence in at least one of these motifs (highlighted in bold in table 1), and the implications of this will be discussed throughout the review. For example, the mitotic kinases Nek6, Nek7, Nek9, Haspin and Bub1 all have non-canonical DFG motifs, although all conserve the aspartic acid (table 1). However, in this case, there is as yet no evidence that these divergent DFG motifs confer a substantial biochemical difference. Indeed, when the divergent leucine of Nek7 was mutated to the more common phenylalanine, the mutant protein exhibited comparable activity to the wild-type protein [39].

At present, there are crystal structures of active and inactive forms of Aurora-A kinase and Plk1. The structure of Haspin has been resolved in an active form, whereas those of Aurora-B and Bub1 are only available for a partially active form. Mitotic kinases with only the inactive structure include Nek2, Nek7, Haspin and Mps1/TTK. The molecular detail obtained from the structures of these kinases in active and inactive conformations, in conjunction with information from biochemical assay methods, have greatly facilitated the elucidation of protein kinase regulatory mechanisms. Mitotic kinases for which there are currently no structures available include Cdk1, Aurora-C, Plk2, Plk3, Nek6, Nek9, Lats1, Lats2, Bub1b and Greatwall.

## 5. Common themes among the regulatory strategies of protein kinases

**Figure 3.**
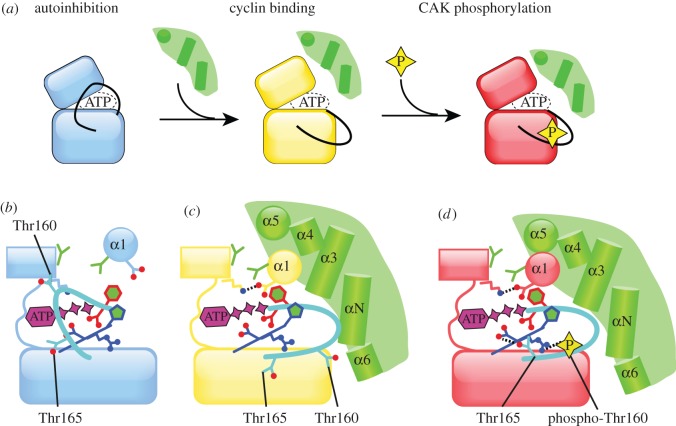
A schematic to summarize Cdk2 activation. (*a*) A simplified scheme showing two-step activation of Cdk2 by Cyclin A binding and CAK phosphorylation. The first step of Cdk2 activation is through binding of a cyclin partner protein, which induces a number of conformational changes, resulting in a partially active kinase that bears many of the hallmarks of an active kinase structure. Cyclin binding also exposes Thr160 within the activation loop to solvent, allowing access to an activating kinase. The second step of Cdk2 activation is phosphorylation on Thr160, by a variety of kinases collectively called Cdk-activating kinase (CAK). (*b*) A schematic of inactive Cdk2 alone (PDB code 1HCK), following the same conventions as figure 2*c*. The structure of Cdk2 appears as if incompletely folded, with many structural elements displaced from their expected positions. (*c*) The structure of Cyclin A-bound Cdk2 (PDB code 1FIN) reveals a partly active conformation, with the Lys–Glu salt bridge in place. Cyclin A is coloured green, with key structural elements that bind Cdk2 labelled. (*d*) The phosphorylated Cdk2/Cyclin A structure (PDB code 1JST) resembles that of active PKA, with all the expected interactions in place.

Looking at mitotic kinases from a structural perspective, it becomes apparent that kinase activation is not a binary process (from inactive to active), but that there are intermediate states that may exhibit varying degrees of activity [66]. We will refer to these as either ‘inactive’ or ‘partially active’ states, because it is difficult to judge how much catalytic activity these states possess, based on structures alone. However, it is straightforward to identify which conformational changes need to occur in order to bring about a fully active conformation, and hence the mechanisms of activation can be inferred. Kinases can adopt a variety of inactive conformations, and the intricate control mechanisms of protein kinases are extremely diverse. Nevertheless, the strategies used to regulate the catalytic activities of a protein can readily be categorized into phosphorylation (and consequently dephosphorylation), autoinhibition and the binding of a partner protein [58,59]. Some kinases may use more than one of these mechanisms in order to change their conformation from an inactive structure to an active structure and thereby achieve full activation. All three mechanisms are used in the regulation of Cdk2, which on its own exists in an inactive, autoinhibited state, achieves partial activity upon binding of Cyclin A and achieves full activity only when this complex is phosphorylated on the activation loop (figure 3) [67–69]. The activation of Cdk2 has been extensively characterized, and it thus serves as a useful basis for the analysis of other kinase activation mechanisms. In this review, we will only discuss aspects of Cdk2 regulation that have clear relevance to the mitotic kinases; other aspects (such as inhibitory proteins) have been reviewed elsewhere [10].

## 6. The structural mechanisms of regulation by autoinhibition

We think it is helpful to draw a distinction between autoinhibited and inactive kinase states because they are potentially different stages in a kinase activation mechanism. We think of autoinhibition as an intramolecular mechanism that blocks the activity of the kinase, as distinct from low activity (or unactivated) states of the kinase or inhibitory events mediated by other factors. From a structural perspective, an autoinhibited kinase exhibits a clearly defined (and ordered) feature that prevents activity. Crucially, there should be a clearly defined physiologically relevant event that antagonizes the autoinhibitory mechanism, as a precursor to activation. Autoinhibition allows kinases to be kept in an inactive state, and thus release is an initial step in the pathway of protein kinase activation. For example, a kinase that is capable of self-activation (through autophosphorylation) may use an autoinhibitory mechanism to prevent inappropriate kinase activity. Another defining feature of an autoinhibited conformation is that it can be reversed through mutation, leading to dramatic increases in kinase activity, and perhaps the best-known example of this is the Val600Glu mutation of BRAF, a driver mutation in cancers such as melanoma [70]. Two typical modes of autoinhibition are the binding of a regulatory domain to the catalytic domain and a conformation of the activation segment that blocks the active site, as exemplified by Src/Abl and BRAF, respectively [70–72].

**Figure 4.**
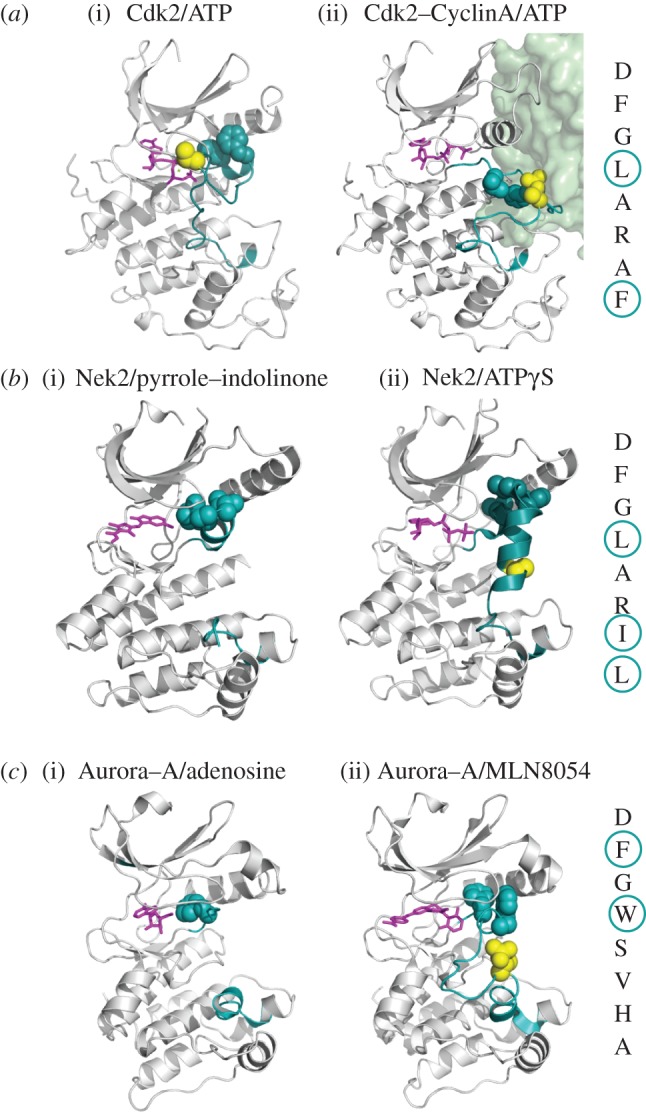
Autoinhibited structures in which the activation segment forms an ordered structure that disrupts the Lys–Glu pair. In each panel, the name of the kinase and the ligand bound to the active site are shown. The activation loop is coloured green–blue, and the Ser/Thr residue that is phosphorylated upon kinase activation is coloured yellow in structures in which it was modelled. At the far right of each panel, the sequence of the DFG motif plus five residues is shown vertically, with hydrophobic residues that block the formation of the Lys–Glu salt bridge circled. (*a*) (i) The autoinhibited conformation of Cdk2 (PDB code 1HCK). (ii) The active conformation of Cdk2-Cyclin A (PDB code 1JST), with the cyclin shown as a green surface. (*b*) Two potential autoinhibited conformations of Nek2: (i) the activation segment is partially disordered (PDB code 2JAV); (ii) the activation segment was fully modelled (PDB code 2W5B). (*c*) Two potential autoinhibited conformations of Aurora-A: (i) the activation segment is partially disordered (PDB code 1MUO); (ii) the activation segment was fully modelled (PDB code 2WTV).

The first crystal structure of Cdk2 revealed an autoinhibited conformation in which the active site is partially blocked by the activation segment (figures 3*b* and 4*a*) [67]. The five residues C-terminal to the DFG motif form a small helix that sits in the active site, and this conformation of the activation segment is incompatible with productive binding of protein substrate, although ATP is able to bind to the active site. The structure exhibits additional features that explain catalytic inactivity—the α1 helix (equivalent to αC of PKA) is displaced from what would be expected in an active kinase structure, there is no Lys–Glu salt bridge, and the hydrophobic spine is interrupted between Leu55 (α1/αC) and Phe146 (DFG motif). The hydroxyl group of Thr160 is buried in the structure, preventing efficient phosphorylation by CAK. This autoinhibitory conformation is relieved through the binding of a cyclin protein, which is described below. There is currently no available structure of Cdk1, although it is probably regulated through a similar mechanism.

Structures of Nek2 bound to a number of different ligands suggest that it too may have an autoinhibited form involving the activation segment. The first structure of Nek2, determined by the groups of Smerdon and Knapp, bound to a pyrrole–indolinone inhibitor, revealed a helical conformation of the DFG motif and five residues that follow that is very similar to the conformation of autoinhibited Cdk2 (figure 4*b*) [73]. Indeed, this feature is conserved between Cdk2 and Neks at the sequence level. Our structure of Nek2 bound to a non-hydrolyzable ATP analogue, which arguably represents a more physiological state of the kinase, shows that the activation segment forms two helices that would prevent binding of substrate peptide, the first of which closely resembles the situation in Cdk2 (figure 4*b*) [74]. In both Nek2 structures, self-activation is prevented because the Lys–Glu salt bridge is blocked by the helix formed from the activation segment. Further structures of Nek2 from our laboratory bound to more than a dozen different ligands indicate that the activation segment of Nek2 is able to adopt many different conformations, and it remains to be discovered which of them, if any, represent a physiological autoinhibitory conformation of the kinase [75–77].

The activation segment of Aurora-A can adopt related inactive conformations in the presence of adenosine, a quinazoline-based inhibitor or the MLN8054 inhibitor (figure 4*c*) [78–80]. A common feature of these three conformations is the interaction between the activation segment and the αC-helix, which blocks the lysine–glutamate salt bridge. When bound to MLN8054, the DFG motif adopts a flipped DFG-up conformation distinct from DFG-out, in which Trp277 packs against Val174 and occupies a very similar position to that observed for Val600 in the BRAF structure, suggesting that this structure might represent an autoinhibitory state. Consistent with this idea, mutation of Val174 to methionine increases kinase activity and has been identified in melanoma as a potential cancer driver mutation [81].

**Figure 5.**
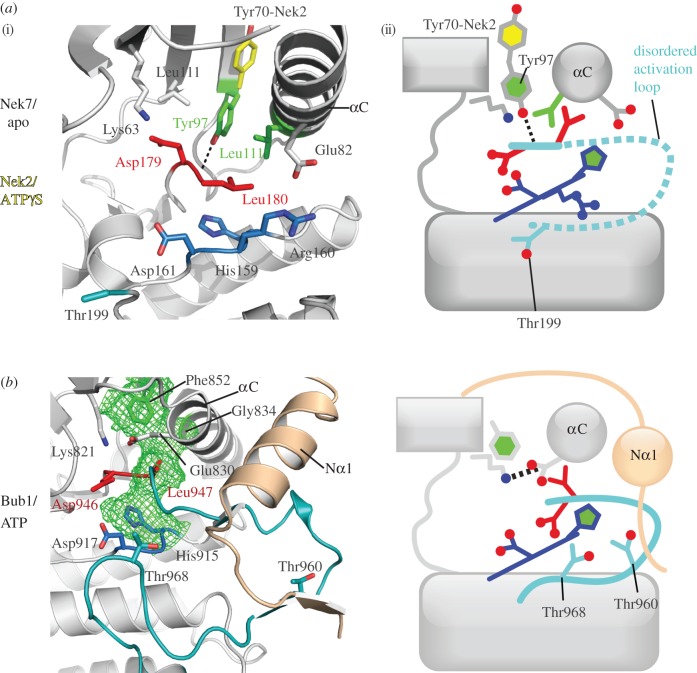
The β4-strand component of the hydrophobic spine is displaced in Nek7 and Bub1. (i) The structures in cartoon representation, with key residues shown as sticks; (ii) the same information in a schematic representation, based on figure 2*c*. (*a*) The structure of Nek7 (PDB code 2WQM) reveals an autoinhibited ‘Tyr-down’ conformation, in which the side chain of Tyr97, the β4-strand component of the hydrophobic spine, points into the active site, which displaces the C-helix from its active position. In order for Nek7 to be catalytically active, Tyr97 must change conformation to adopt the position shown for the equivalent side chain in Nek2 (Tyr70, yellow, PDB code 2W5B). (*b*) The structure of Bub1 (PDB code 3E7E) reveals a hydrophobic spine comprising only three side chains (green mesh). Phe852 (which is equivalent to Tyr97 on Nek7) adopts the down position, but this does not disrupt the Lys–Glu pair, and a continuous hydrophobic spine is formed (green mesh). This is because the side chain of Phe852 fills the void that is left by the absence of a side chain on the αC component of the spine (Gly834). The extended N-terminus of the Bub1 kinase domain (beige), which is crucial for activity, interacts with the activation segment. Thr960 and Thr968 are candidates to form interactions with Asp917 in the active conformation.

There is evidence for a mechanistically distinct form of autoinhibition in Nek kinases. In Nek2, Nek6, Nek7 and Nek9, the residue on the β4-strand that would be expected to form part of the hydrophobic spine in the active conformation is a tyrosine residue (Tyr70^Nek2^, Tyr108^Nek6^ and Tyr97^Nek7^ and Tyr104^Nek9^). In our Nek7 structure, this residue appears to be crucial in coordinating the key residues within the active site and holding the kinase in the inactive state (figure 5*a*) [39]. The crystal structure of Nek7 reveals that Tyr97 is held in the ‘down’ conformation and hydrogen bonds to Leu180 of its non-canonical DLG motif. The aromatic ring of the tyrosine is sandwiched between the Leu111 of the hinge region and Leu86, preventing formation of the salt bridge between Lys63 and Glu82, which is essential for catalysis [39]. Mutation of Tyr97^Nek7^ to Ala increases kinase activity and leads to loss of cell-cycle regulation. We postulated that autoinhibition is reversed by association with the non-catalytic domain of Nek9, which can enhance the activity of wild-type Nek7 but not the Tyr97Ala mutant. As Nek6 is so similar in domain structure and homology to Nek7, it is predicted that Nek6 possesses an equivalent inhibitory tyrosine residue, and indeed mutation of Tyr108^Nek6^ increases kinase activity. Autoinhibition of Nek6 and Nek7 is necessary for maintaining low levels of activity of these mitotic kinases during interphase [39], and inappropriately active kinase leads to cell death. An autoinhibitory role for the tyrosine residue of Nek2 or Nek9 has not yet been examined, but the aromatic side chain of Tyr70^Nek2^ can flip down into the active site in the presence of some ATP-competitive inhibitors, in a way that closely resembles the autoinhibitory mechanism of Nek7 [39,76].

**Figure 6.**
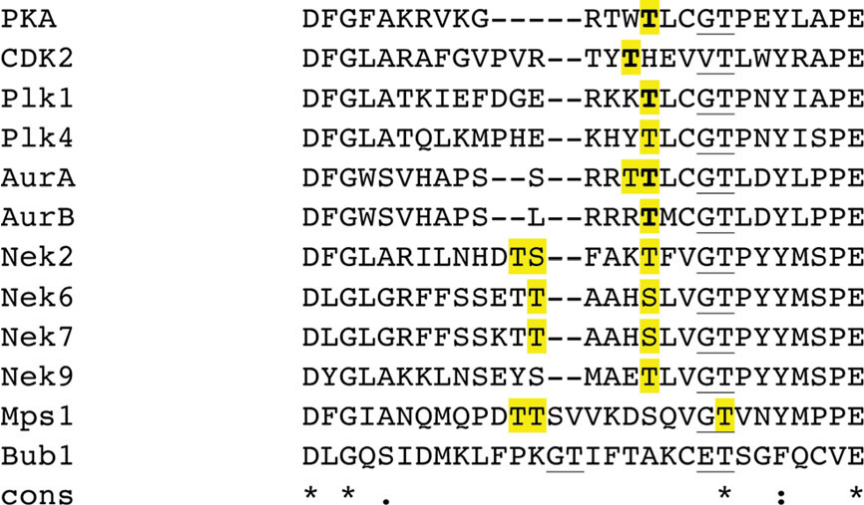
Sequence alignment of mitotic kinase activation segments, compared with protein kinase A (PKA). The alignment was produced using ClustalW2. Absolutely conserved residues are marked with an asterisk, conservatively substituted residues with a colon and semi-conservative substitutions with a dot. Potential sites of activating phosphorylation are highlighted in yellow. Sites that have been confirmed to have an activating role in a crystal structure are marked in bold type. The ‘GT’ motif in the P+1 loop is underlined.

The structure of Bub1, resolved by the Yu group, has many of the hallmarks of an active kinase, but exhibits a number of unusual features, including a non-canonical hydrophobic spine and a potentially autoinhibitory conformation of the activation segment incompatible with substrate protein binding (figure 5*b*) [82]. The αC-helix is shifted compared with other kinases, although the lysine–glutamate pair remains intact. Coupled with this, there is a radical alteration to the hydrophobic spine: the usual hydrophobic spine position on the αC-helix is occupied by Gly834, and the β4-strand residue (Phe852) appears to compensate for this by being in the ‘down’ position to form a three-residue spine instead of the usual four. The activation segment of Bub1 is well ordered, but adopts a conformation that would be expected to block substrate access to the active sites and hence may be autoinhibitory. Asp917 of the non-canonical HRD motif (of sequence HGD) does not interact with a threonine, and in the paper that describes the structure, Thr960 is described as the likely partner because it lies within a canonical GT motif. Thr960 is about 20 Å away from Asp917 and is buried, suggesting that a major conformational change would be required to produce the fully active conformation of the activation segment. However, we think that Thr968 is a more likely candidate to interact with Asp917, even though it is not in a canonical GT motif and is instead preceded by a glutamic acid. Thr968 is only 9 Å away from Asp917, and it lies in the expected position five residues N-terminal to the non-canonical APE motif of Bub1 (actual sequence CVE, which sits at the equivalent position in the structure, at the start of αEF; figures 5 and 6). Indeed, mutation of Thr968 to alanine dramatically reduced kinase activity, consistent with its role in forming an interaction with the catalytic Asp917 residue. If this were the case, a relatively small change would be required to produce a conformation more optimal for substrate binding, which might simply occur by an induced-fit mechanism upon binding of the substrate. Further biochemical and structural studies on Bub1 will be required to clarify this matter.

## 7. The structural mechanisms of regulation by phosphorylation on the activation loop

Many protein kinases require phosphorylation for activation, at one or multiple serine, threonine and tyrosine residues. Although phosphorylation can occur at sites throughout the protein, the region that is most commonly involved in regulation is the activation loop. Phosphorylation here is often a key characteristic of protein kinase regulation and is frequently used as a marker of kinase activity. The activation loops of kinases vary in length and often contain multiple serine, threonine or tyrosine residues, making it difficult to predict the sites of activating phosphorylation based purely on sequence (figure 6). Phosphorylation sites can be identified by mass spectrometry, which are then validated, most commonly using site-specific mutagenesis. In principle, mutating the site of activating phosphorylation to alanine should render the kinase inactive (or at least decrease activity), and mutating to aspartate/glutamate should activate the kinase (at least to higher than the activity of unphosphorylated kinase). In practice, this can yield false results because serine/threonine residues can play structural or catalytic roles in the kinase mechanism (e.g. the conserved threonine in the P + 1 loop) and aspartate/glutamate can be a poor phosphomimic [83]. Of course, these ambiguities are resolved if you can obtain a crystal structure of the kinase phosphorylated specifically on the appropriate residue that shows how the modification enhances activity. This can be technically challenging, and has thus far only been done for Plk1, Aurora-A and Aurora-B of the mitotic kinases [84–86]. These structures provide a mechanistic basis for understanding the role of phosphorylation in the activation of mitotic kinases.

Kinases that are activated through phosphorylation of their activation loop usually have a canonical HRD motif, and the arginine residue within the motif recognizes the phosphorylated activation loop [60]. This interaction stabilizes the activation segment in a conformation that efficiently recognizes substrate. The HRD motif is thus crucial in maintenance of the kinase active state as well as being important in catalysis. In many kinases, the phosphorylated side chain forms interact with other arginine/lysine residues that also stabilize the kinase in its active conformation, such as in the β9 strand of the activation segment or in the αC-helix. In the absence of phosphorylation, these basic side chains repel each other, destabilizing the active kinase conformation. The presence or absence of the residues that recognize the primary phosphorylation site reveal whether or not a kinase is likely to be regulated by phosphorylation on the activation loop.

Phosphorylation of Cdk2/Cyclin A complex is essential for full activity, although because CAK is constitutively active, this is not a regulatory step in the strictest sense. In the structure of the phosphorylated Cdk2/Cyclin A complex (figure 4*a*), pThr160 is mostly buried, where it interacts with three arginine residues at the canonical positions (αC, β9, HRD).

**Figure 7.**
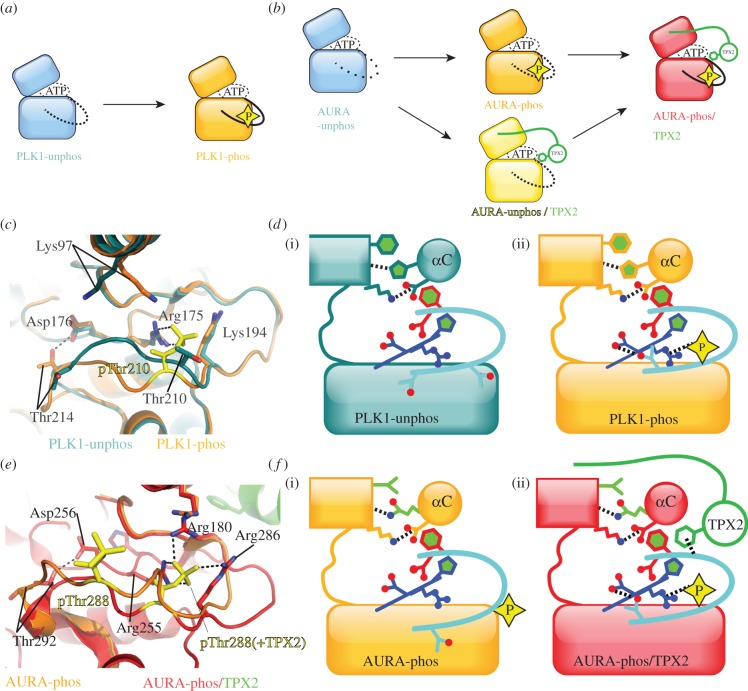
The mechanism of Plk1 and Aurora-A activation is mainly through stabilization of the activation segment conformation. (*a*) Summary of the one-step activation mechanism of Plk1, based on the crystal structures in unphosphorylated state (PLK1-unphos, blue, PDB code 3D5U) and phosphorylated state (PLK1-phos, orange, PDB code 3D5W). Upon phosphorylation, the activation loop changes conformation from an inactive position inconsistent with substrate protein binding (dashed line) to an active position (solid line). (*b*) Summary of the two-step activation mechanism of Aurora-A, based on crystal structures of the unphosphorylated state (AURA-unphos, blue, PDB codes 1MUO, 1MQ4), phosphorylated state (AURA-phos, orange, PDB code 1OL7) and phosphorylated state in complex with TPX2 (AURA-phos/TPX2, red, PDB code 1OL5). Note that there is currently no crystal structure of unphosphorylated Aurora-A bound to TPX2 (yellow). The activation segment of unphosphorylated Aurora-A is partially disordered; the activation segment of phosphorylated Aurora-A is ordered, but in an inactive conformation incompatible with substrate protein binding; and the addition of TPX2 locks the activation segment into a conformation compatible with protein substrate binding. (*c*) Superposed crystal structures of PLK1-phos (orange) and PLK1-unphos (blue), shown in the vicinity of the activation segment. Phosphorylated Thr210 is shown in yellow, and key interactions are shown as dashed lines. Conformational differences are mostly restricted to the activation segment. In addition, the side chain of Lys97 also moves closer to the phosphorylated form of Thr210, although the functional significance of this is unclear. (*d*) Schematic diagrams summarizing the key features of (i) PLK1-unphos and (ii) PLK1-phos. Note that the unphosphorylated Plk1 structure has many of the features of an active kinase, with the exception of the activation loop conformation. (*e*) Superposed crystal structures of phosphorylated Aurora-A alone (orange) and bound to TPX2 aa1–43 (red). Phosphorylated Thr288 is shown in yellow, and the position of pThr288 in the presence of TPX2 is labelled (+TPX2). Key interactions are shown as dashed lines. The side chain of Arg286 is only shown in the TPX2-bound form of Aurora-A because, if it were to be shown in the other structure, its position would obscure the interactions of pThr288. (*f*) Schematic summarizing the key features of (i) AURA-phos and (ii) AURA-phos/TPX2. Note that the AURA-phos structure bears a close resemblance to PLK1-unphos, and AURA-phos/TPX2 is similar to PLK1-phos.

There is ongoing debate regarding the kinases upstream of Plk1, which include Aurora-A, Aurora-B, Plkk1 (Plk kinase-1, also known as Snf1 in humans) and PKA [12,87–90]. The structural mechanism of Plk1 activation is more straightforward—its conformation is largely unchanged during the activation process and appears to be regulated only by phosphorylation (figure 7). During mitosis, and more specifically at the G2/M phase boundary, Plk1 undergoes phosphorylation at Thr210 in the activation loop [91,92]. Structures resolved by the Romanowski group show that this threonine residue moves a distance of 2.9 Å upon phosphorylation (PDB entries 3D5U, 3D5W) [84]. The structure of unphosphorylated Plk1 has many of the features of an active kinase, including an intact hydrophobic spine and the Lys–Glu salt bridge, and structural changes upon phosphorylation are largely confined to the activation segment. It is tempting to speculate that an additional H-bond interaction between His105, part of the hydrophobic spine, and the β4 strand main chain contributes to the stability of the unphosphorylated Plk1 structure (figure 7). The phosphate group on Thr210 interacts with the side chain of Arg175 in the HRD motif, but Plk1 lacks basic residues on the canonical positions of the αC helix and β9, and so this interaction appears to be sufficient to lock the activation segment in the active conformation, such that Thr214 can form an H-bond with the catalytic aspartic acid of the HRD motif. Plk1 does have basic residues at other positions that are in proximity to the phosphate group (figure 7*c* and table 1). Hence, the presence of the phosphate group neutralizes the positive charge of this region, in addition to promoting stabilizing interactions.

Aurora-A and Aurora-B kinase are phosphorylated at threonine residues within their activation loops at Thr288 and Thr232, respectively, which increases catalytic activity [85,86]. Although Aurora-A and Aurora-B can be phosphorylated by other kinases, the principal mechanism in cells appears to be autophosphorylation [90,93,94]. Like Plk1, the structure of unphosphorylated Aurora-A has an intact hydrophobic spine and Lys–Glu salt bridge, suggesting that its activation occurs primarily through stabilization of the activation segment. Aurora kinases have canonical basic residues for recognition of phosphate in the HRD motif and the αC helix, but, like Plk1, lack a canonical basic residue on the β9 strand, which is perhaps compensated for by the presence of an additional basic residue on the activation loop. Remarkably, despite the presence of these three basic residues, our structure of phosphorylated Aurora-A shows that the activation segment does not adopt a conformation similar to that observed for active kinase structures—the phosphate group on Thr288 does not interact with Arg255 of the HRD motif, and Thr292 does not form the expected H-bond with Asp256 in the HRD motif (figure 7*e*,*f*). The final stabilization of the activation segment is brought about by binding of TPX2, which will be described in the following section. There is no structure of phosphorylated Aurora-B alone, so it is not clear whether phosphorylation is sufficient to order the activation segment. Aurora-A has also been reported to be activated by phosphorylation at multiple sites within its N-terminal domain, which has been observed as an autoinhibitory domain [95–97]. However, there are currently no structural data regarding this domain, nor how it might regulate catalytic activity.

Among the mitotic Nek kinases, there are no structures of Nek6 and Nek9, and there are only unphosphorylated structures of Nek2 and Nek7. However, these structures are sufficient to infer some details of their regulation by phosphorylation. Structures of Nek2 and Nek7 show disrupted hydrophobic spines and disordered activation segments (or activation segments in inactive conformations), and the Lys–Glu salt bridge is absent. These kinases need to undergo substantial changes in order to form an active conformation, and although we cannot at present say whether phosphorylation is sufficient to induce all of these changes, we know that phosphorylation is a key determinant of activity in all these kinases. Nek2 is activated upon autophosphorylation of Thr175 within the activation loop [32,73], which is reversed by PP1 [98–100]. Nek2 is also phosphorylated by the signalling protein Mst2 in its C-terminal non-catalytic domain, although the mechanism by which this step activates the kinase is unknown [101]. The activation loops of Nek6 and Nek7 are rich in Ser/Thr residues, although mutagenesis studies have highlighted the importance of some, but not others (figure 6). These kinases are capable of autophosphorylation at Ser206^Nek6^ and Ser195^Nek7^, respectively [102], and Nek9 can also phosphorylate them at these serine residues [31,38]. Nek9 is phosphorylated by Plk1 on Thr210 [103], but can also autophosphorylate this threonine [31,104]. The activating phosphorylation sites on Nek2, Nek6, Nek7 and Nek9 are equivalent to Thr210^Plk1^/Thr288^Aurora-A^, and they all have at least two basic residues in the canonical positions for phosphate recognition that could stabilize an active conformation. Therefore, we assume that phosphorylation would lead to stabilization of the activation segment in a conformation similar to that observed for PKA.

**Figure 8.**
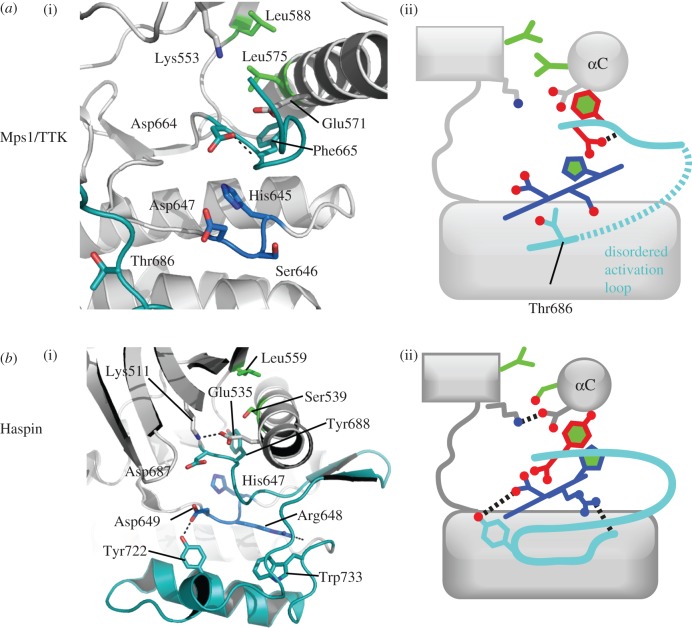
Structures of Mps1/TTK and Haspin. (*a*) Structure of Mps1/TTK captured in an inactive conformation (PDB code 2ZMC). (i) The structure viewed in the vicinity of the activation segment (light blue). (ii) The key features are summarized in the schematic illustration. (*b*) Structure of Haspin in an active conformation, in the absence of phosphorylation or any other external factor (PDB code 2VUW). (i) The structure viewed in the vicinity of the activation segment (light blue), which adopts a highly unusual structure. (ii) Key residues and interactions are summarized.

The phospho-regulation of Mps1 is complex and has several interesting features. Mps1 is capable of autophosphorylating multiple residues within its activation loop, and Thr675, Thr676 and Thr686 are all phosphorylated during mitosis [55,105,106]. There are no structures of Mps1 in an active conformation, and many structures in similar inactive conformations from the Eyers, Tabernero, Knapp, Lei and Liu groups (figure 8*a*) [105,107–109]. The lysine–glutamate pair is not formed, and instead the side chain of Glu571 interacts with the main chain of the activation segment, and the side chain of Lys553 is bound to a polyethylene glycol molecule originating from the crystallization solution. The hydrophobic spine is disrupted between Phe665 of the DFG motif and His645 of the HRD motif. The mechanism by which activation loop phosphorylation of Mps1 activates the kinase has not been resolved, but it must be highly unusual because Mps1 lacks the usual motifs that might interact with the phosphorylated activation loop. It is not an RD kinase (the Arg is replaced by Ser), the two basic residues on the αC-helix are positioned on the opposite side of the helix to the activation loop, and there is no basic residue in the β9 strand. There is a basic residue (Lys681) at the equivalent position on the activation loop to that involved in phosphate recognition in Plk1/Aurora-A/Aurora-B; however, this residue is disordered in all current Mps1 structures, and its role in kinase activation is unknown (figure 6). It has been proposed that a basic patch C-terminal to the activation segment might interact with the phosphorylated activation loop, and indeed mutations at Lys708 and Lys710 reduce kinase activity [105]. However, the only crystal structure of Mps1 in which the phosphorylated side chains of Thr675, Thr676 and Ser677 are ordered also shows an inactive conformation, with all three phosphates positioned out into solvent (PDB code 3H9F). In this case, an inactive conformation of the activation loop is probably stabilized by interactions with the ligand bound to the active site [109]. More surprisingly, there is strong evidence that Thr686 within the GT motif is phosphorylated during mitosis and that this form of the kinase is active. This is an unprecedented modification in a protein kinase, and suggests that this modification might switch the specificity from serine/threonine to tyrosine substrates [105]. Structural studies on specific phospho-states of Mps1 will clarify the mechanism of activation, but will be technically challenging.

## 8. The structural mechanisms of regulation by binding partners

Cdks are the best-known examples of protein kinases that are activated by the binding of a partner protein, which in this case are cyclins, and the details of the mechanism have been extensively reviewed [9,10]. Cyclin A forms an extensive interface with unphosphorylated Cdk2, interacting primarily with the α1/αC helix and activation segment (figures 3 and 4*a*). This releases the activation segment from its autoinhibitory conformation, and remoulds the conformation of Cdk2 into a partially active form, in which the Lys–Glu salt bridge is formed, the hydrophobic spine is assembled and Thr160 is accessible for phosphorylation. One way of thinking about the function of the cyclin is that it completes the proper folding of the kinase domain, and that the Cdk on its own has an incomplete catalytic domain because it requires the cyclin for it to fold properly and have activity [10].

**Figure 9.**
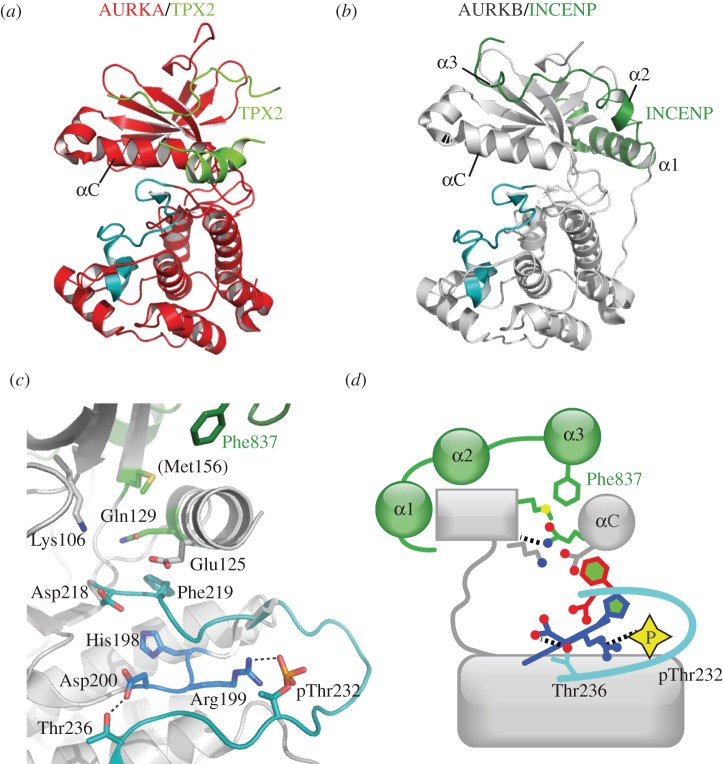
Comparison of activation mechanisms of Aurora-A and Aurora-B. (*a*) Crystal structure of phosphorylated Aurora-A (red, activation segment in green–blue) bound to TPX2 (green), based on PDB code 1OL5. (*b*) Structure of phosphorylated Aurora-B (grey, activation segment in green–blue) bound to INCENP (green), based on PDB code 2BFX. The three α-helices of the INCENP fragment are labelled α1, α2 and α3. (*c*) Structure of INCENP/Aurora-B complex in the vicinity of the activation segment. It is not known whether INCENP affects the conformation of the activation segment, which it does not directly contact. It is thought that Phe837 of INCENP causes a rotation of the αC helix that prevents Lys–Glu salt bridge formation. Note that *Xenopus laevis* Aurora-B has residue Met156, equivalent to human Leu140; all the other residues are labelled with human protein numbering. (*d*) Schematic illustration of the INCENP/Aurora-B complex.

The catalytic activity of Aurora kinases depends on the binding of a number of protein-binding partners and crystal structures of the Aurora-A/TPX2 and Aurora-B/inner centromere protein (INCENP) complexes have shed light on their respective activation mechanisms (figures 7 and 9) [85,86]. TPX2 is a microtubule-associated protein [110], and INCENP is another component of the CPC [111]. Residues 7–21 of TPX2 bind to the N-lobe of Aurora-A in an extended conformation, and residues 30–40 form a short helix that binds between the N-lobe and the activation loop (figure 9*a*) [85]. This interaction generates conformational changes in the activation loop of Aurora-A, leading to a fully active conformation, in which key interactions are established, between pThr288 and Arg255, Arg180 (αC-helix) and Arg286, as well as between Thr292 and Asp256 (figure 7*e*,*f*). When TPX2 is bound to Aurora-A, the phosphate group on Thr288 is buried, whereas without TPX2, this phosphate is accessible to phosphatases (figure 7*e*). This explains why dephosphorylation of Aurora-A by PP1 is less efficient in the presence of TPX2.

The structure of Aurora-B bound to INCENP, from the Musacchio group, shows a similar conformation of the activation segment to that observed in the Aurora-A/TPX2 complex (figure 9). Residues 798–841 of INCENP bind to the N-lobe of Aurora-B, forming a series of three helices, the final two of which occupy a site similar to that of residues 7–21 of TPX2 [86]. The first helix of INCENP does not resemble TPX2, but instead wraps around the other side of the N-lobe, forming a contact with the C-terminal region of Aurora-B. The crystal structure does not capture a fully active conformation of Aurora-B, because the αC-helix and C-lobe are both rotated 15° away from what is found in the structure of Aurora-A/TPX2, and one consequence of this is that the Lys–Glu salt bridge is not formed. It has been proposed that phosphorylation of a TSS motif in INCENP changes the position of Phe837, allowing the αC-helix to rotate into its active position. Aurora-A and -B are more than 70 per cent identical in their catalytic domains. TPX2 can discriminate between Aurora-A and -B on the basis of a single residue, Gly198, which is Asn in Aurora-B [112]. Indeed, a Gly198 to Asn mutation in Aurora-A not only ablates TPX2 binding, but confers regulation by INCENP, so that the mutant somewhat resembles Aurora-B in its cellular behaviour [113,114].

More recently, the contribution of TPX2 binding and phosphorylation to Aurora-A activity have been thoroughly explored. TPX2 can stimulate the activity of unphosphorylated Aurora-A, and its energetic contribution to catalysis is 2.1–2.3 kcal mol^−1^, independent of the phosphorylation state of Aurora-A [66]. Our working model is that TPX2 activates unphosphorylated Aurora-A through partial stabilization of the activation segment. Indeed, we postulate that the first step of Aurora-A activation is partial stabilization of the activation segment, whether by phosphorylation or by protein partner binding. This model is in contrast to the activation pathway of Cdks, in which cyclin binding and phosphorylation have distinct and sequential roles.

## 9. Mitotic kinases with constitutively active catalytic domains

Bub1 and Haspin do not appear to be obviously regulated through any of the three common mechanisms discussed above. This is perhaps not surprising because they diverge hugely in their regulatory motifs (table 1). Although the structure of Bub1 was previously mentioned as an example of autoinhibition, this assignment is debatable and it could well be regarded as an example of a constitutively active kinase, with a highly unusual activation segment.

The primary sequence of Haspin is divergent from other protein kinases, including the catalytic and regulatory motifs (table 1). Crystal structures from the Knapp and Musacchio laboratories revealed a recognizable kinase fold, albeit highly modified, with three major differences from that adopted by other kinases (figure 8*b*) [115,116]. First, the N-lobe is larger, with elements such as an α-helix inserted between β4 and β5 that stabilizes the Gly-rich loop. Second, the activation segment is highly divergent and lacks an APE motif. Third, the C-lobe is modified by the addition of a two-strand β-sheet that interacts with the loop between αC and β4, and by the deletion of the αG helix, which usually abuts the activation segment. The structures appear to show a constitutively active kinase, with most of the key catalytic elements such as the Lys–Glu salt bridge in place, albeit with a few interesting twists. The αC component of the hydrophobic spine is a Ser, and the void is partly filled by water molecules and partly by the side chain of Tyr from the DYT motif that replaces DFG at the start of the activation segment. The impact of this on Mg/ATP binding is unclear, although it has been suggested that, unlike in other kinases, ATP might be recognized directly by the catalytic Asp of the HRD motif and a histidine residue close by. The activation segment is fully ordered in a conformation that is highly divergent from other active kinases, but which appears to be consistent with substrate binding. The activation loop has no GT motif, and instead the Tyr722 side chain forms a hydrogen bond with the catalytic Asp649. During mitosis, Haspin is highly phosphorylated, but the structures suggest that Haspin is probably not regulated by phosphorylation on the activation loop, even though it conserves two out of three canonical residues involved in phosphate recognition (Arg648 of the HRD motif and Arg692 on β9). Indeed, both basic side chains form salt bridges with glutamic acid residues, and hence their positive charges are already neutralized and they fulfil structural roles in the absence of phosphorylation. In addition, the side chain of Arg648 forms interactions with the main chain at Trp733 and Glu735. Furthermore, there is only one suitable phosphorylatable residue on the activation segment (Ser705), which has not been observed to be phosphorylated [115].

## 10. Summary and outstanding questions

During mitosis, there is a peak in cellular phosphorylation, which is thought to effect a wholesale transformation in the activities of cellular proteins [117]. These phosphorylation events are catalysed by a subset of protein kinases that are activated during mitosis. Recent structural studies have provided molecular insights into the activation mechanisms of these kinases. Structural studies provide essential validation of key regulatory steps and contribute to the eventual aim of understanding these pathways at a systems level; and yet there are many questions left unresolved. For a start, there are no structures of key mitotic kinases such as Lats kinases or Greatwall, and even in the case of kinases with numerous entries in the Protein Data Bank, there remain many mechanistic questions that require a structural biology approach. In this concluding section of the review, we will highlight some of the outstanding questions that need to be addressed by structural biologists who study mitotic kinases.

Protein crystallography has revealed that active protein kinases adopt a specific conformation with respect to a key set of features, including the lysine–glutamate pair, the HRD and DFG motifs, the hydrophobic spine and a set of three basic residues that coordinate the activating phosphate group. These features are mostly conserved at the sequence level in mitotic protein kinases, although all mitotic kinases are divergent in at least one feature, which influences how they achieve an active conformation. Of the mitotic kinases, only Aurora-A, Plk1 and Haspin have been crystallized in fully active conformations, although the structures of Aurora-B/INCENP and Bub1 do exhibit most of the features of an active kinase. Perhaps most strikingly of all, structures of Haspin and Bub1 show how an active kinase conformation (or almost active) can be achieved with highly divergent kinases despite lacking one of the key hydrophobic spine residues (Bub1) or several of the usual motifs (Haspin). In crystal structures of kinases in inactive conformations, these features are displaced from their active positions in a variety of ways, which have illuminated their mechanisms of regulation; for example, in the identification of a novel mechanism of autoinhibition in Nek7 and explaining why phosphorylation is insufficient for full activity in Aurora-A. Other structures raise the prospect of the existence of autoinhibited states (Nek2, Aurora-A) or highlight the requirement for further conformational changes to achieve full activity (Aurora-B/INCENP, Bub1).

### 10.1. Autoinhibition and other mechanisms that prevent activity

Mechanisms that prevent the untimely activation of kinases are crucial to ensure the fidelity of cell division. Autoinhibition is one such mechanism, as used in the regulation of kinases such as Cdk2 and BRAF. In this review, we have summarized the evidence for autoinhibitory conformations of mitotic kinases based on crystal structures of Aurora-A, Nek2 and Nek7. Further studies on these kinases are required to determine the contribution that these autoinhibitory states make to the physiological regulation of kinase activity. Future structural studies will reveal how these autoinhibitory states are reversed, for example through the interaction of Nek9 with Nek7. Reversal of activating phosphorylation by phosphatases is another mechanism deployed to prevent untimely kinase activation, and a clear example of this is the inhibition of Nek2 by PP1 [100]. It is important to identify the *bona fide* phosphatase partners of other mitotic kinases, to understand the basis of their selectivity and regulation. Along these lines, it has recently been shown that PP6, not PP1, is the key Aurora-A inhibitory phosphatase and is capable of dephosphorylating the TPX2/Aurora-A complex [118]. A clear priority for future studies is the elucidation of the structural basis by which cellular phosphatases recognize mitotic kinases.

### 10.2. Activation by phosphorylation

Almost all mitotic kinases are regulated at some level by phosphorylation, and in many cases a site of activating phosphorylation has been identified on the activation loop (table 1). In Plk1, this phosphorylation event is sufficient to generate a stable conformation of the activation loop compatible with substrate binding, but Aurora-A also requires the binding of TPX2. How this works in other mitotic kinases could be resolved by tackling the crystal structures of these kinases in singly phosphorylated forms without other binding partners. However, this is technically challenging, and is unlikely to be high on the list of priorities of structural biologists unless there is strong reason to believe that the answer will be interesting. We think, though, that the effort would be worth it for Mps1 because it lacks the three canonical basic residues used for recognition of a phosphorylated activation loop, and so phosphorylation might act through a novel structural mechanism. In fact, we believe that it is feasible to derive many insights into the conformational changes upon phosphorylation from biochemical data, which might be a more attractive option to address these issues [66]. Perhaps a more important challenge for structural biologists is to resolve the structural basis of kinase regulation by phosphorylation at sites away from the activation loop in the catalytic domain, such as inhibitory phosphorylation of Ser342 of Aurora-A or Ser241 of Nek2 [73,83]. Generating protein samples for structural biology (or biochemical studies) that have a fully specified and homogeneous phosphorylation state remains a challenge, and we are currently exploring chemical approaches to solve this problem.

### 10.3. Role of non-catalytic domains and binding partners

Some mitotic kinases do not become fully activated until bound to a partner protein, triggering a conformational change, which may release an autoinhibitory state (such as postulated in Nek9 allosteric activation of Nek7), reveal previously hidden additional phosphorylation sites (such as in cyclin activation of Cdks) or lock the protein in an active state (such as in TPX2 stimulation of phospho-Aurora-A activity). From a structural point of view, there are broad similarities between the ways that cyclins remodel the Cdk structure and TPX2/INCENP complement the catalytic domains of Aurora kinases. Not least of these is that the binding partners form crucial interactions with the α1/αC helix, and (at least in the case of cyclins and TPX2) with the activation segment. In this regard, they function similarly to the N- and C-terminal extensions of PKA [85]. To date, all structures of activatory binding partners show recognition of a hydrophobic groove on the surface of the N-lobe, which is formed by the αC helix and β4 strand. This feature is commonly involved in the regulation of AGC family kinases, to which the Auroras bear closest resemblance, but not in other kinase families [119]. Other mitotic kinases, including Plk1, Nek2 and Mps1, also have a hydrophobic groove, and although we do not know of any proteins that interact at this site in these kinases, we have observed crystal packing interactions with the hydrophobic groove in Nek2 that mimic the situation in AGC kinases, and it is known that dimerization activates the Nek2 kinase in cells [74]. Although in existing structures Nek7 lacks an obvious hydrophobic groove, Tyr97 is located precisely where one might expect a groove to open up to accommodate Nek9 in order to reverse autoinhibition, and this hypothesis has informed our current studies on this complex. There is clearly a lot more to kinase regulation and function than is encoded in the structure of the catalytic domain, and further examples of domains and binding partners that ‘complete’ the kinase domain are undoubtedly out there waiting to be discovered. Indeed, many other activatory binding partners of Aurora-A have been identified, such as Ajuba, I2 and Cep192, and it is not known whether they work through mechanisms similar to or different from TPX2. Activation by a binding partner localizes kinase activity to a specific spindle region or protein complex. This fulfils an important regulatory role because the consensus sequences phosphorylated by some mitotic kinases overlap, perhaps best exemplified by Aurora-B (which phosphorylates substrates at the kinetochore) and Aurora-A (which phosphorylates substrates at the centrosome and spindle microtubules), but both kinases have the capability to phosphorylate peptides with similar sequences *in vitro* [120]. It would not be surprising if the other mitotic kinase families used localization through activatory binding partners to determine substrate specificity.

### 10.4. Substrate recognition

There are currently no structures of mitotic kinase catalytic domains bound to substrates, and although in some cases these interactions can be modelled with a degree of confidence, there are other examples for which an experimental structure would be very interesting [120]. For example, Haspin and Bub1 have unusual structures in the expected substrate-binding regions, and in the particular case of Haspin, it would be interesting to resolve how its unusual activation segment contributes to the reading and writing of the histone code. Moreover, the structure of Bub1 bound to a peptide substrate would address whether or not a substantial conformational change is required in the activation segment in order to form an active structure. Sequences outside the catalytic domain may contribute to substrate recognition, and the best-known example of this is Plk1, which recognizes substrates through its polo box [91,92]. There are countless other examples for which we have no structural information, such as the interaction between the non-catalytic domain of Aurora-A and p53, the recognition of substrates by the N-terminal extensions of Nek6 and Nek7, and the contribution of Haspin's non-catalytic domain to substrate recognition [121]. These are important targets for future structural studies, although challenging, because these domains appear to be natively disordered in the absence of substrate.

### 10.5. Mitotic kinases as drug targets

Mitotic kinases are an important class of drug target, and understanding their regulatory mechanisms is key to effective new treatments for various hyperproliferative diseases, most notably cancer. Structural characterization of kinases facilitates rational drug design, enabling kinase active sites to be selectively targeted by tailor-made inhibitors. This has played a crucial role in the development of ATP-competitive inhibitors of Aurora kinases, Plk1, Mps1 and Nek2 [77,108,109,122–124]. Building on these studies, with the aid of structural biology, it should be possible to design chemical inhibitors specific for each and every mitotic kinase. In combination with systems biology approaches, this could transform our understanding of the networks of kinases that coordinate mitosis. It will require some thoughtful experimental design to dissect out the role of different populations of each kinase as mitosis progresses, and to tease apart their interdependent relationships. Moreover, this will have to include the contribution of kinase-binding partners such as TPX2, which is not only crucial for the localization and activity of Aurora-A, but can affect the potency of kinase inhibitors [79,125].

In this review, we have described the remarkable progress in the resolution of mitotic kinase structures, and explained how these models have contributed to our understanding of their regulation and function. There are still many unanswered questions relating to the structural biology of mitotic kinases, and we have identified what we consider to be priorities for future work. Finally, we look forward to further developments in the field as the molecular details of mitotic signalling pathways are elaborated further, and this knowledge is translated into improvements in human health.

## 11. Acknowledgements

R.B. is a Royal Society Research Fellow and acknowledges grant support from Cancer Research UK (C24461/A12772 and C24461/A13231). T.H. and S.Y. are supported by the University of Leicester. A.F. acknowledges grant support from The Wellcome Trust and the Association for International Cancer Research (AICR).

## References

[1] Musacchio A Salmon ED 2007 The spindle-assembly checkpoint in space and time. Nat. Rev. Mol. Cell. Biol. 8, 379–393 10.1038/nrm2163 (doi:10.1038/nrm2163) 17426725

[2] Lengauer C Kinzler KW Vogelstein B 1997 Genetic instability in colorectal cancers. Nature 386, 623–627 10.1038/386623a0 (doi:10.1038/386623a0) 9121588

[3] Nigg EA 2002 Centrosome aberrations: cause or consequence of cancer progression? Nat. Rev. Cancer 2, 815–825 10.1038/nrc924 (doi:10.1038/nrc924) 12415252

[4] Kops GJ Weaver BA Cleveland DW 2005 On the road to cancer: aneuploidy and the mitotic checkpoint. Nat. Rev. Cancer 5, 773–785 10.1038/nrc1714 (doi:10.1038/nrc1714) 16195750

[5] Manchado E Guillamot M Malumbres M 2012 Killing cells by targeting mitosis. Cell Death Differ. 19, 369–377 10.1038/cdd.2011.197 (doi:10.1038/cdd.2011.197) 22223105 PMC3278741

[6] Ma HT Poon RY 2011 How protein kinases co-ordinate mitosis in animal cells. Biochem. J. 435, 17–31 10.1042/BJ20100284 (doi:10.1042/BJ20100284) 21406064

[7] Manning G Whyte DB Martinez R Hunter T Sudarsanam S 2002 The protein kinase complement of the human genome. Science 298, 1912–1934 10.1126/science.1075762 (doi:10.1126/science.1075762) 12471243

[8] Hunt T 1989 Maturation promoting factor, cyclin and the control of M-phase. Curr. Opin. Cell Biol. 1, 268–274 10.1016/0955-0674(89)90099-9 (doi:10.1016/0955-0674(89)90099-9) 2576632

[9] Morgan DO 1995 Principles of CDK regulation. Nature 374, 131–134 10.1038/374131a0 (doi:10.1038/374131a0) 7877684

[10] Pavletich NP 1999 Mechanisms of cyclin-dependent kinase regulation: structures of Cdks, their cyclin activators, and Cip and INK4 inhibitors. J. Mol. Biol. 287, 821–828 10.1006/jmbi.1999.2640 (doi:10.1006/jmbi.1999.2640) 10222191

[11] Enserink JM Kolodner RD 2010 An overview of Cdk1-controlled targets and processes. Cell Div. 5, 11 10.1186/1747-1028-5-11 (doi:10.1186/1747-1028-5-11) 20465793 PMC2876151

[12] Barr FA Sillje HH Nigg EA 2004 Polo-like kinases and the orchestration of cell division. Nat. Rev. Mol. Cell. Biol. 5, 429–440 10.1038/nrm1401 (doi:10.1038/nrm1401) 15173822

[13] Glover DM 2005 Polo kinase and progression through M phase in *Drosophila*: a perspective from the spindle poles. Oncogene 24, 230–237 10.1038/sj.onc.1208279 (doi:10.1038/sj.onc.1208279) 15640838

[14] Lowery DM Lim D Yaffe MB 2005 Structure and function of Polo-like kinases. Oncogene 24, 248–259 10.1038/sj.onc.1208280 (doi:10.1038/sj.onc.1208280) 15640840

[15] Archambault V Carmena M 2012 Polo-like kinase-activating kinases: aurora A, Aurora B and what else? Cell Cycle. 11, 1490–1495 10.4161/cc.19724 (doi:10.4161/cc.19724) 22433949 PMC3341226

[16] Lindon C Pines J 2004 Ordered proteolysis in anaphase inactivates Plk1 to contribute to proper mitotic exit in human cells. J. Cell Biol. 164, 233–241 10.1083/jcb.200309035 (doi:10.1083/jcb.200309035) 14734534 PMC2172335

[17] Chan CS Botstein D 1993 Isolation and characterization of chromosome-gain and increase-in-ploidy mutants in yeast. Genetics 135, 677–691 8293973 10.1093/genetics/135.3.677 PMC1205712

[18] Francisco L Chan CS 1994 Regulation of yeast chromosome segregation by Ipl1 protein kinase and type 1 protein phosphatase. Cell Mol. Biol. Res. 40, 207–213 7874197

[19] Barr AR Gergely F 2007 Aurora-A: the maker and breaker of spindle poles. J. Cell Sci. 120, 2987–2996 10.1242/jcs.013136 (doi:10.1242/jcs.013136) 17715155

[20] Nikonova AS Astsaturov I Serebriiskii IG Dunbrack RL Jr Golemis EA In press. Aurora A kinase (AURKA) in normal and pathological cell division. Cell Mol. Life Sci. 10.1007/s00018-012-1073-7 (doi:10.1007/s00018-012-1073-7) PMC3607959 22864622

[21] Mori D Yamada M Mimori-Kiyosue Y Shirai Y Suzuki A Ohno S Saya H Wynshaw-Boris A Hirotsune S 2009 An essential role of the aPKC-Aurora A-NDEL1 pathway in neurite elongation by modulation of microtubule dynamics. Nat. Cell. Biol. 11, 1057–1068 10.1038/ncb1919 (doi:10.1038/ncb1919) 19668197

[22] Pugacheva EN Jablonski SA Hartman TR Henske EP Golemis EA 2007 HEF1-dependent Aurora A activation induces disassembly of the primary cilium. Cell 129, 1351–1363 10.1016/j.cell.2007.04.035 (doi:10.1016/j.cell.2007.04.035) 17604723 PMC2504417

[23] Goto H Yasui Y Nigg EA Inagaki M 2002 Aurora-B phosphorylates histone H3 at serine28 with regard to the mitotic chromosome condensation. Genes Cells 7, 11–17 10.1046/j.1356-9597.2001.00498.x (doi:10.1046/j.1356-9597.2001.00498.x) 11856369

[24] Lampson MA Cheeseman IM 2011 Sensing centromere tension: aurora B and the regulation of kinetochore function. Trends Cell Biol. 21, 133–140 10.1016/j.tcb.2010.10.007 (doi:10.1016/j.tcb.2010.10.007) 21106376 PMC3049846

[25] Sasai K 2004 Aurora-C kinase is a novel chromosomal passenger protein that can complement Aurora-B kinase function in mitotic cells. Cell Motil. Cytoskeleton 59, 249–263 10.1002/cm.20039 (doi:10.1002/cm.20039) 15499654

[26] Carmena M Earnshaw WC 2003 The cellular geography of aurora kinases. Nat. Rev. Mol. Cell. Biol. 4, 842–854 10.1038/nrm1245 (doi:10.1038/nrm1245) 14625535

[27] Morris NR 1975 Mitotic mutants of *Aspergillus nidulans*. Genet. Res. 26, 237–254 10.1017/S0016672300016049 (doi:10.1017/S0016672300016049) 773766

[28] Morris NR Osmani SA Engle DB Doonan JH 1989 The genetic analysis of mitosis in *Aspergillus nidulans*. Bioessays 10, 196–201 10.1002/bies.950100605 (doi:10.1002/bies.950100605) 2662965

[29] O'Regan L Blot J Fry AM 2007 Mitotic regulation by NIMA-related kinases. Cell Div. 2, 25 10.1186/1747-1028-2-25 (doi:10.1186/1747-1028-2-25) 17727698 PMC2018689

[30] Fry AM Arnaud L Nigg EA 1999 Activity of the human centrosomal kinase, Nek2, depends on an unusual leucine zipper dimerization motif. J. Biol. Chem. 274, 16 304–16 310 10.1074/jbc.274.23.16304 (doi:10.1074/jbc.274.23.16304) 10347187

[31] Roig J Mikhailov A Belham C Avruch J 2002 Nercc1, a mammalian NIMA-family kinase, binds the Ran GTPase and regulates mitotic progression. Genes Dev. 16, 1640–1658 10.1101/gad.972202 (doi:10.1101/gad.972202) 12101123 PMC186374

[32] Fry AM Schultz SJ Bartek J Nigg EA 1995 Substrate specificity and cell cycle regulation of the Nek2 protein kinase, a potential human homolog of the mitotic regulator NIMA of *Aspergillus nidulans*. J. Biol. Chem. 270, 12 899–12 905 10.1074/jbc.270.21.12899 (doi:10.1074/jbc.270.21.12899) 7759549

[33] Lu KP Osmani SA Means AR 1993 Properties and regulation of the cell cycle-specific NIMA protein kinase of *Aspergillus nidulans*. J. Biol. Chem. 268, 8769–8776 8473320

[34] Fry AM 2002 The Nek2 protein kinase: a novel regulator of centrosome structure. Oncogene 21, 6184–6194 10.1038/sj.onc.1205711 (doi:10.1038/sj.onc.1205711) 12214248

[35] Hames RS Wattam SL Yamano H Bacchieri R Fry AM 2001 APC/C-mediated destruction of the centrosomal kinase Nek2A occurs in early mitosis and depends upon a cyclin A-type D-box. EMBO J. 20, 7117–7127 10.1093/emboj/20.24.7117 (doi:10.1093/emboj/20.24.7117) 11742988 PMC125337

[36] Hayes MJ Kimata Y Wattam SL Lindon C Mao G Yamano H Fry AM 2006 Early mitotic degradation of Nek2A depends on Cdc20-independent interaction with the APC/C. Nat. Cell. Biol. 8, 607–614 10.1038/ncb1410 (doi:10.1038/ncb1410) 16648845

[37] Sdelci S Bertran MT Roig J 2011 Nek9, Nek6, Nek7 and the separation of centrosomes. Cell Cycle 10, 3816–3817 10.4161/cc.10.22.18226 (doi:10.4161/cc.10.22.18226) 22064517

[38] Belham C Roig J Caldwell JA Aoyama Y Kemp BE Comb M Avruch J 2003 A mitotic cascade of NIMA family kinases. Nercc1/Nek9 activates the Nek6 and Nek7 kinases. J. Biol. Chem. 278, 34 897–34 909 10.1074/jbc.M303663200 (doi:10.1074/jbc.M303663200) 12840024

[39] Richards MW O'Regan L Mas-Droux C Blot JM Cheung J Hoelder S Fry AM Bayliss R 2009 An autoinhibitory tyrosine motif in the cell-cycle-regulated Nek7 kinase is released through binding of Nek9. Mol. Cell. 36, 560–570 10.1016/j.molcel.2009.09.038 (doi:10.1016/j.molcel.2009.09.038) 19941817 PMC2807034

[40] Rapley J Nicolas M Groen A Regue L Bertran MT Caelles C Avruch J Roig J 2008 The NIMA-family kinase Nek6 phosphorylates the kinesin Eg5 at a novel site necessary for mitotic spindle formation. J. Cell Sci. 121, 3912–3921 10.1242/jcs.035360 (doi:10.1242/jcs.035360) 19001501 PMC4066659

[41] Sdelci S Schutz M Pinyol R Bertran MT Regue L Caelles C Vernos I Roig J 2012 Nek9 Phosphorylation of NEDD1/GCP-WD contributes to Plk1 control of gamma-tubulin recruitment to the mitotic centrosome. Curr. Biol. 22, 1516–1523 10.1016/j.cub.2012.06.027 (doi:10.1016/j.cub.2012.06.027) 22818914

[42] Logarinho E Bousbaa H 2008 Kinetochore-microtubule interactions ‘in check’ by Bub1, Bub3 and BubR1: the dual task of attaching and signalling. Cell Cycle 7, 1763–1768 (doi:10.4161/cc.7.12.6180) 18594200 10.4161/cc.7.12.6180

[43] Elowe S. 2011 Bub1 and BubR1: at the interface between chromosome attachment and the spindle checkpoint. Mol. Cell. Biol. 31, 3085–3093 10.1128/mcb.05326-11 (doi:10.1128/mcb.05326-11) 21628528 PMC3147602

[44] Bolanos-Garcia VM Blundell TL 2011 BUB1 and BUBR1: multifaceted kinases of the cell cycle. Trends Biochem. Sci. 36, 141–150 10.1016/j.tibs.2010.08.004 (doi:10.1016/j.tibs.2010.08.004) 20888775 PMC3061984

[45] Bolanos-Garcia VM 2009 The crystal structure of the N-terminal region of BUB1 provides insight into the mechanism of BUB1 recruitment to kinetochores. Structure 17, 105–116 10.1016/j.str.2008.10.015 (doi:10.1016/j.str.2008.10.015) 19141287 PMC2683949

[46] Bolanos-Garcia VM 2011 Structure of a Blinkin-BUBR1 complex reveals an interaction crucial for kinetochore-mitotic checkpoint regulation via an unanticipated binding site. Structure 19, 1691–1700 10.1016/j.str.2011.09.017 (doi:10.1016/j.str.2011.09.017) 22000412 PMC3267040

[47] Larsen NA Al-Bassam J Wei RR Harrison SC 2007 Structural analysis of Bub3 interactions in the mitotic spindle checkpoint. Proc. Natl Acad. Sci. USA 104, 1201–1206 10.1073/pnas.0610358104 (doi:10.1073/pnas.0610358104) 17227844 PMC1770893

[48] Higgins JM 2010 Haspin: a newly discovered regulator of mitotic chromosome behavior. Chromosoma 119, 137–147 10.1007/s00412-009-0250-4 (doi:10.1007/s00412-009-0250-4) 19997740 PMC2839057

[49] Kelly AE Ghenoiu C Xue JZ Zierhut C Kimura H Funabiki H 2010 Survivin reads phosphorylated histone H3 threonine 3 to activate the mitotic kinase Aurora B. Science 330, 235–239 10.1126/science.1189505 (doi:10.1126/science.1189505) 20705815 PMC3177562

[50] Wang F Dai J Daum JR Niedzialkowska E Banerjee B Stukenberg PT Gorbsky GJ Higgins JM 2010 Histone H3 Thr-3 phosphorylation by Haspin positions Aurora B at centromeres in mitosis. Science 330, 231–235 10.1126/science.1189435 (doi:10.1126/science.1189435) 20705812 PMC2967368

[51] Wang F Ulyanova NP van der Waal MS Patnaik D Lens SM Higgins JM 2011 A positive feedback loop involving Haspin and Aurora B promotes CPC accumulation at centromeres in mitosis. Curr. Biol. 21, 1061–1069 10.1016/j.cub.2011.05.016 (doi:10.1016/j.cub.2011.05.016) 21658950 PMC3118923

[52] Liu X Winey M 2012 The MPS1 family of protein kinases. Annu. Rev. Biochem. 81, 561–585 10.1146/annurev-biochem-061611-090435 (doi:10.1146/annurev-biochem-061611-090435) 22482908 PMC4026297

[53] Lan W Cleveland DW 2010 A chemical tool box defines mitotic and interphase roles for Mps1 kinase. J. Cell Biol. 190, 21–24 10.1083/jcb.201006080 (doi:10.1083/jcb.201006080) 20624898 PMC2911672

[54] Lee S Thebault P Freschi L Beaufils S Blundell TL Landry CR Bolanos-Garcia VM Elowe S 2012 Characterization of spindle checkpoint kinase Mps1 reveals domain with functional and structural similarities to tetratricopeptide repeat motifs of Bub1 and BubR1 checkpoint kinases. J. Biol. Chem. 287, 5988–6001 10.1074/jbc.M111.307355 (doi:10.1074/jbc.M111.307355) 22187426 PMC3285366

[55] Tyler RK Chu ML Johnson H McKenzie EA Gaskell SJ Eyers PA 2009 Phosphoregulation of human Mps1 kinase. Biochem J. 417, 173–181 10.1042/bj20081310 (doi:10.1042/bj20081310) 18680479

[56] Cui Y Cheng X Zhang C Zhang Y Li S Wang C Guadagno TM 2010 Degradation of the human mitotic checkpoint kinase Mps1 is cell cycle-regulated by APC-cCdc20 and APC-cCdh1 ubiquitin ligases. J. Biol. Chem. 285, 32 988–32 998 10.1074/jbc.M110.140905 (doi:10.1074/jbc.M110.140905) PMC2963336 20729194

[57] Knighton DR Zheng JH Ten Eyck LF Ashford VA Xuong NH Taylor SS Sowadski JM 1991 Crystal structure of the catalytic subunit of cyclic adenosine monophosphate-dependent protein kinase. Science 253, 407–414 10.1126/science.1862342 (doi:10.1126/science.1862342) 1862342

[58] Huse M Kuriyan J 2002 The conformational plasticity of protein kinases. Cell 109, 275–282 10.1016/S0092-8674(02)00741-9 (doi:10.1016/S0092-8674(02)00741-9) 12015977

[59] Johnson LN Noble ME Owen DJ 1996 Active and inactive protein kinases: structural basis for regulation. Cell 85, 149–158 10.1016/S0092-8674(00)81092-2 (doi:10.1016/S0092-8674(00)81092-2) 8612268

[60] Nolen B Taylor S Ghosh G 2004 Regulation of protein kinases; controlling activity through activation segment conformation. Mol. Cell. 15, 661–675 10.1016/j.molcel.2004.08.024 (doi:10.1016/j.molcel.2004.08.024) 15350212

[61] Lowe ED Noble ME Skamnaki VT Oikonomakos NG Owen DJ Johnson LN 1997 The crystal structure of a phosphorylase kinase peptide substrate complex: kinase substrate recognition. EMBO J. 16, 6646–6658 10.1093/emboj/16.22.6646 (doi:10.1093/emboj/16.22.6646) 9362479 PMC1170269

[62] Parang K Till JH Ablooglu AJ Kohanski RA Hubbard SR Cole PA 2001 Mechanism-based design of a protein kinase inhibitor. Nat. Struct. Biol. 8, 37–41 10.1038/83028 (doi:10.1038/83028) 11135668

[63] Gibbs CS Zoller MJ 1991 Rational scanning mutagenesis of a protein kinase identifies functional regions involved in catalysis and substrate interactions. J. Biol. Chem. 266, 8923–8931 2026604

[64] Schindler T Bornmann W Pellicena P Miller WT Clarkson B Kuriyan J 2000 Structural mechanism for STI-571 inhibition of abelson tyrosine kinase. Science 289, 1938–1942 10.1126/science.289.5486.1938 (doi:10.1126/science.289.5486.1938) 10988075

[65] Kornev AP Haste NM Taylor SS Eyck LF 2006 Surface comparison of active and inactive protein kinases identifies a conserved activation mechanism. Proc. Natl Acad. Sci. USA 103, 17 783–17 788 10.1073/pnas.0607656103 (doi:10.1073/pnas.0607656103) PMC1693824 17095602

[66] Dodson CA Bayliss R 2012 Activation of Aurora-A kinase by protein partner binding and phosphorylation are independent and synergistic. J. Biol. Chem. 287, 1150–1157 10.1074/jbc.M111.312090 (doi:10.1074/jbc.M111.312090) 22094468 PMC3256853

[67] De Bondt HL Rosenblatt J Jancarik J Jones HD Morgan DO Kim SH 1993 Crystal structure of cyclin-dependent kinase 2. Nature 363, 595–602 10.1038/363595a0 (doi:10.1038/363595a0) 8510751

[68] Jeffrey PD Russo AA Polyak K Gibbs E Hurwitz J Massague J Pavletich NP 1995 Mechanism of CDK activation revealed by the structure of a cyclinA-CDK2 complex. Nature 376, 313–320 10.1038/376313a0 (10.1038/376313a0) 7630397

[69] Russo AA Jeffrey PD Pavletich NP 1996 Structural basis of cyclin-dependent kinase activation by phosphorylation. Nat. Struct. Biol. 3, 696–700 10.1038/nsb0896-696 (doi:10.1038/nsb0896-696) 8756328

[70] Wan PT 2004 Mechanism of activation of the RAF-ERK signaling pathway by oncogenic mutations of B-RAF. Cell 116, 855–867 10.1016/S0092-8674(04)00215-6 (doi:10.1016/S0092-8674(04)00215-6) 15035987

[71] Xu W Doshi A Lei M Eck MJ Harrison SC 1999 Crystal structures of c-Src reveal features of its autoinhibitory mechanism. Mol. Cell. 3, 629–638 10.1016/S1097-2765(00)80356-1 (doi:10.1016/S1097-2765(00)80356-1) 10360179

[72] Nagar B Hantschel O Young MA Scheffzek K Veach D Bornmann W Clarkson B Superti-Furga G Kuriyan J 2003 Structural basis for the autoinhibition of c-Abl tyrosine kinase. Cell 112, 859–871 10.1016/S0092-8674(03)00194-6 (doi:10.1016/S0092-8674(03)00194-6) 12654251

[73] Rellos P 2007 Structure and regulation of the human Nek2 centrosomal kinase. J. Biol. Chem. 282, 6833–6842 10.1074/jbc.M609721200 (doi:10.1074/jbc.M609721200) 17197699

[74] Westwood I Cheary DM Baxter JE Richards MW van Montfort RL Fry AM Bayliss R 2009 Insights into the conformational variability and regulation of human Nek2 kinase. J. Mol. Biol. 386, 476–485 10.1016/j.jmb.2008.12.033 (doi:10.1016/j.jmb.2008.12.033) 19124027 PMC2741569

[75] Solanki S Innocenti P Mas-Droux C Boxall K Barillari C van Montfort RL Aherne GW Bayliss R Hoelder S 2011 Benzimidazole inhibitors induce a DFG-out conformation of never in mitosis gene A-related kinase 2 (Nek2) without binding to the back pocket and reveal a nonlinear structure-activity relationship. J. Med. Chem. 54, 1626–1639 10.1021/jm1011726 (doi:10.1021/jm1011726) 21366329

[76] Whelligan DK 2010 Aminopyrazine inhibitors binding to an unusual inactive conformation of the mitotic kinase Nek2: SAR and structural characterization. J. Med. Chem. 53, 7682–7698 10.1021/jm1008727 (doi:10.1021/jm1008727) 20936789 PMC2972649

[77] Innocenti P 2012 Design of potent and selective hybrid inhibitors of the mitotic kinase Nek2: structure–activity relationship, structural biology, and cellular activity. J. Med. Chem. 55, 3228–3241 10.1021/jm201683b (doi:10.1021/jm201683b) 22404346 PMC3935458

[78] Cheetham GM Knegtel RM Coll JT Renwick SB Swenson L Weber P Lippke JA Austen DA 2002 Crystal structure of aurora-2, an oncogenic serine/threonine kinase. J. Biol. Chem. 277, 42 419–42 422 10.1074/jbc.C200426200 (doi:10.1074/jbc.C200426200) 12237287

[79] Dodson CA Kosmopoulou M Richards MW Atrash B Bavetsias V Blagg J Bayliss R 2010 Crystal structure of an Aurora-A mutant that mimics Aurora-B bound to MLN8054: insights into selectivity and drug design. Biochem. J. 427, 19–28 10.1042/BJ20091530 (doi:10.1042/BJ20091530) 20067443

[80] Heron NM 2006 SAR and inhibitor complex structure determination of a novel class of potent and specific Aurora kinase inhibitors. Bioorg. Med. Chem. Lett. 16, 1320–1323 10.1016/j.bmcl.2005.11.053 (doi:10.1016/j.bmcl.2005.11.053) 16337122

[81] Bibby RA Tang C Faisal A Drosopoulos K Lubbe S Houlston R Bayliss R Linardopoulos S 2009 A cancer-associated aurora A mutant is mislocalized and misregulated due to loss of interaction with TPX2. J. Biol. Chem. 284, 33 177–33 184 10.1074/jbc.M109.032722 (doi:10.1074/jbc.M109.032722) PMC2785160 19801554

[82] Kang J Yang M Li B Qi W Zhang C Shokat KM Tomchick DR Machius M Yu H 2008 Structure and substrate recruitment of the human spindle checkpoint kinase Bub1. Mol. Cell. 32, 394–405 10.1016/j.molcel.2008.09.017 (doi:10.1016/j.molcel.2008.09.017) 18995837 PMC2644263

[83] Littlepage LE Wu H Andresson T Deaneham JK Amundadottir LT Ruderman JV 2002 Identification of phosphorylated residues that affect the activity of the mitotic kinase Aurora-A. Proc. Natl Acad. Sci. USA 99, 15 440–15 445 10.1073/pnas.202606599 (doi:10.1073/pnas.202606599) PMC137735 12422018

[84] Elling RA Fucini RV Romanowski MJ 2008 Structures of the wild-type and activated catalytic domains of *Brachydanio rerio* Polo-like kinase 1 (Plk1): changes in the active-site conformation and interactions with ligands. Acta Crystallogr. D Biol. Crystallogr. 64, 909–918 10.1107/S0907444908019513 (doi:10.1107/S0907444908019513) 18703838

[85] Bayliss R Sardon T Vernos I Conti E 2003 Structural basis of Aurora-A activation by TPX2 at the mitotic spindle. Mol. Cell 12, 851–862 10.1016/S1097-2765(03)00392-7 (doi:10.1016/S1097-2765(03)00392-7) 14580337

[86] Sessa F Mapelli M Ciferri C Tarricone C Areces LB Schneider TR Stukenberg PT Musacchio A 2005 Mechanism of Aurora B activation by INCENP and inhibition by hesperadin. Mol. Cell 18, 379–391 10.1016/j.molcel.2005.03.031 (doi:10.1016/j.molcel.2005.03.031) 15866179

[87] Qian YW Erikson E Maller JL 1998 Purification and cloning of a protein kinase that phosphorylates and activates the polo-like kinase Plx1. Science 282, 1701–1704 10.1126/science.282.5394.1701 (doi:10.1126/science.282.5394.1701) 9831560

[88] Ellinger-Ziegelbauer H Karasuyama H Yamada E Tsujikawa K Todokoro K Nishida E 2000 Ste20-like kinase (SLK), a regulatory kinase for polo-like kinase (Plk) during the G2/M transition in somatic cells. Genes Cells 5, 491–498 10.1046/j.1365-2443.2000.00337.x (doi:10.1046/j.1365-2443.2000.00337.x) 10886374

[89] Kelm O Wind M Lehmann WD Nigg EA 2002 Cell cycle-regulated phosphorylation of the Xenopus polo-like kinase Plx1. J. Biol. Chem. 277, 25 247–25 256 10.1074/jbc.M202855200 (doi:10.1074/jbc.M202855200) 11994303

[90] Scutt PJ Chu ML Sloane DA Cherry M Bignell CR Williams DH Eyers PA 2009 Discovery and exploitation of inhibitor-resistant aurora and polo kinase mutants for the analysis of mitotic networks. J. Biol. Chem. 284, 15 880–15 893 10.1074/jbc.M109.005694 (doi:10.1074/jbc.M109.005694) PMC2708884 19359241

[91] Elia AE 2003 The molecular basis for phosphodependent substrate targeting and regulation of Plks by the Polo-box domain. Cell 115, 83–95 10.1016/S0092-8674(03)00725-6 (doi:10.1016/S0092-8674(03)00725-6) 14532005

[92] Cheng KY Lowe ED Sinclair J Nigg EA Johnson LN 2003 The crystal structure of the human polo-like kinase-1 polo box domain and its phospho-peptide complex. EMBO J. 22, 5757–5768 10.1093/emboj/cdg558 (doi:10.1093/emboj/cdg558) 14592974 PMC275415

[93] Yasui Y 2004 Autophosphorylation of a newly identified site of Aurora-B is indispensable for cytokinesis. J. Biol. Chem. 279, 12 997–13 003 10.1074/jbc.M311128200 (doi:10.1074/jbc.M311128200) 14722118

[94] Jelluma N Brenkman AB van den Broek NJ Cruijsen CW van Osch MH Lens SM Medema RH Kops GJ 2008 Mps1 phosphorylates Borealin to control Aurora B activity and chromosome alignment. Cell 132, 233–246 10.1016/j.cell.2007.11.046 (doi:10.1016/j.cell.2007.11.046) 18243099

[95] Plotnikova OV Nikonova AS Loskutov YV Kozyulina PY Pugacheva EN Golemis EA 2012 Calmodulin activation of Aurora-A kinase (AURKA) is required during ciliary disassembly and in mitosis. Mol. Biol. Cell. 23, 2658–2670 10.1091/mbc.E11-12-1056 (doi:10.1091/mbc.E11-12-1056) 22621899 PMC3395655

[96] Reboutier D Troadec MB Cremet JY Fukasawa K Prigent C 2012 Nucleophosmin/B23 activates Aurora A at the centrosome through phosphorylation of serine 89. J. Cell Biol. 197, 19–26 10.1083/jcb.201107134 (doi:10.1083/jcb.201107134) 22451695 PMC3317798

[97] Zhang Y Ni J Huang Q Ren W Yu L Zhao S 2007 Identification of the auto-inhibitory domains of Aurora-A kinase. Biochem. Biophys. Res. Commun. 357, 347–352 10.1016/j.bbrc.2007.03.129 (doi:10.1016/j.bbrc.2007.03.129) 17433255

[98] Helps NR Luo X Barker HM Cohen PT 2000 NIMA-related kinase 2 (Nek2), a cell-cycle-regulated protein kinase localized to centrosomes, is complexed to protein phosphatase 1. Biochem. J. 349, 509–518 10.1042/0264-6021:3490509 (doi:10.1042/0264-6021:3490509) 10880350 PMC1221174

[99] Mi J Guo C Brautigan DL Larner JM 2007 Protein phosphatase-1alpha regulates centrosome splitting through Nek2. Cancer Res. 67, 1082–1089 10.1158/0008-5472.CAN-06-3071 (doi:10.1158/0008-5472.CAN-06-3071) 17283141

[100] Eto M Elliott E Prickett TD Brautigan DL 2002 Inhibitor-2 regulates protein phosphatase-1 complexed with NimA-related kinase to induce centrosome separation. J. Biol. Chem. 277, 44 013–44 020 10.1074/jbc.M208035200 (doi:10.1074/jbc.M208035200) 12221103

[101] Mardin BR Lange C Baxter JE Hardy T Scholz SR Fry AM Schiebel E 2010 Components of the Hippo pathway cooperate with Nek2 kinase to regulate centrosome disjunction. Nat. Cell. Biol. 12, 1166–1176 10.1038/ncb2120 (doi:10.1038/ncb2120) 21076410 PMC3939356

[102] O'Regan L Fry AM 2009 The Nek6 and Nek7 protein kinases are required for robust mitotic spindle formation and cytokinesis. Mol. Cell. Biol. 29, 3975–3990 10.1128/MCB.01867-08 (doi:10.1128/MCB.01867-08) 19414596 PMC2704745

[103] Bertran MT Sdelci S Regue L Avruch J Caelles C Roig J 2011 Nek9 is a Plk1-activated kinase that controls early centrosome separation through Nek6/7 and Eg5. EMBO J. 30, 2634–2647 10.1038/emboj.2011.179 (doi:10.1038/emboj.2011.179) 21642957 PMC3155310

[104] Regue L Sdelci S Bertran MT Caelles C Reverter D Roig J 2011 DYNLL/LC8 protein controls signal transduction through the Nek9/Nek6 signaling module by regulating Nek6 binding to Nek9. J. Biol. Chem. 286, 18 118–18 129 10.1074/jbc.M110.209080 (doi:10.1074/jbc.M110.209080) PMC3093884 21454704

[105] Wang W 2009 Structural and mechanistic insights into Mps1 kinase activation. J. Cell Mol. Med. 13, 1679–1694 10.1111/j.1582-4934.2008.00605.x (doi:10.1111/j.1582-4934.2008.00605.x) 19120698 PMC2829362

[106] Kang J Chen Y Zhao Y Yu H 2007 Autophosphorylation-dependent activation of human Mps1 is required for the spindle checkpoint. Proc. Natl Acad. Sci. USA 104, 20 232–20237 10.1073/pnas.0710519105 (doi:10.1073/pnas.0710519105) PMC2154414 18083840

[107] Chu ML Chavas LM Douglas KT Eyers PA Tabernero L 2008 Crystal structure of the catalytic domain of the mitotic checkpoint kinase Mps1 in complex with SP600125. J. Biol. Chem. 283, 21 495–21 500 10.1074/jbc.M803026200 (doi:10.1074/jbc.M803026200) 18480048

[108] Chu ML 2010 Biophysical and X-ray crystallographic analysis of Mps1 kinase inhibitor complexes. Biochemistry 49, 1689–1701 10.1021/bi901970c (doi:10.1021/bi901970c) 20099905

[109] Kwiatkowski N 2010 Small-molecule kinase inhibitors provide insight into Mps1 cell cycle function. Nat. Chem. Biol. 6, 359–368 10.1038/nchembio.345 (doi:10.1038/nchembio.345) 20383151 PMC2857554

[110] Heidebrecht HJ Buck F Steinmann J Sprenger R Wacker HH Parwaresch R 1997 p100: a novel proliferation-associated nuclear protein specifically restricted to cell cycle phases S, G2, and M. Blood 90, 226–233 9207457

[111] Earnshaw WC Cooke CA 1991 Analysis of the distribution of the INCENPs throughout mitosis reveals the existence of a pathway of structural changes in the chromosomes during metaphase and early events in cleavage furrow formation. J. Cell Sci. 98, 443–461 1860899 10.1242/jcs.98.4.443

[112] Bayliss R Sardon T Ebert J Lindner D Vernos I Conti E 2004 Determinants for Aurora-A activation and Aurora-B discrimination by TPX2. Cell Cycle 3, 404–407 10.4161/cc.3.4.777 (doi:10.4161/cc.3.4.777) 14752279

[113] Fu J Bian M Liu J Jiang Q Zhang C 2009 A single amino acid change converts Aurora-A into Aurora-B-like kinase in terms of partner specificity and cellular function. Proc. Natl Acad. Sci. USA 106, 6939–6944 10.1073/pnas.0900833106 (doi:10.1073/pnas.0900833106) 19357306 PMC2678431

[114] Hans F Skoufias DA Dimitrov S Margolis RL 2009 Molecular distinctions between Aurora A and B: a single residue change transforms Aurora A into correctly localized and functional Aurora B. Mol. Biol. Cell 20, 3491–3502 10.1091/mbc.E09-05-0370 (doi:10.1091/mbc.E09-05-0370) 19494039 PMC2719567

[115] Eswaran J Patnaik D Filippakopoulos P Wang F Stein RL Murray JW Higgins JM Knapp S 2009 Structure and functional characterization of the atypical human kinase haspin. Proc. Natl Acad. Sci. USA 106, 20 198–20 203 10.1073/pnas.0901989106 (doi:10.1073/pnas.0901989106) PMC2777956 19918057

[116] Villa F Capasso P Tortorici M Forneris F de Marco A Mattevi A Musacchio A 2009 Crystal structure of the catalytic domain of Haspin, an atypical kinase implicated in chromatin organization. Proc. Natl Acad. Sci. USA 106, 20 204–20 209 10.1073/pnas.0908485106 (doi:10.1073/pnas.0908485106) PMC2777964 19918049

[117] Olsen JV 2010 Quantitative phosphoproteomics reveals widespread full phosphorylation site occupancy during mitosis. Sci. Signal. 3, ra3 10.1126/scisignal.2000475 (doi:10.1126/scisignal.2000475) 20068231

[118] Zeng K Bastos RN Barr FA Gruneberg U 2010 Protein phosphatase 6 regulates mitotic spindle formation by controlling the T-loop phosphorylation state of Aurora A bound to its activator TPX2. J. Cell Biol. 191, 1315–1332 10.1083/jcb.201008106 (doi:10.1083/jcb.201008106) 21187329 PMC3010072

[119] Gold MG Barford D Komander D 2006 Lining the pockets of kinases and phosphatases. Curr. Opin. Struct. Biol. 16, 693–701 10.1016/j.sbi.2006.10.006 (doi:10.1016/j.sbi.2006.10.006) 17084073

[120] Alexander J 2011 Spatial exclusivity combined with positive and negative selection of phosphorylation motifs is the basis for context-dependent mitotic signaling. Sci. Signal. 4, ra42 10.1126/scisignal.2001796 (doi:10.1126/scisignal.2001796) 21712545 PMC3939359

[121] Vaz Meirelles G Ferreira Lanza DC da Silva JC Santana Bernachi J Paes Leme AF Kobarg J 2010 Characterization of hNek6 interactome reveals an important role for its short N-terminal domain and colocalization with proteins at the centrosome. J. Proteome Res. 9, 6298–6316 10.1021/pr100562w (doi:10.1021/pr100562w) 20873783

[122] Bouloc N 2010 Structure-based design of imidazo[1,2-a]pyrazine derivatives as selective inhibitors of Aurora-A kinase in cells. Bioorg. Med. Chem. Lett. 20, 5988–5993 10.1016/j.bmcl.2010.08.091 (doi:10.1016/j.bmcl.2010.08.091) 20833547

[123] Yan A Wang L Xu S Xu J 2011 Aurora-A kinase inhibitor scaffolds and binding modes. Drug Discov. Today 16, 260–269 10.1016/j.drudis.2010.12.003 (doi:10.1016/j.drudis.2010.12.003) 21147253

[124] Kothe M Kohls D Low S Coli R Rennie GR Feru F Kuhn C Ding YH 2007 Selectivity-determining residues in Plk1. Chem. Biol. Drug Des. 70, 540–546 10.1111/j.1747-0285.2007.00594.x (doi:10.1111/j.1747-0285.2007.00594.x) 18005335

[125] Anderson K 2007 Binding of TPX2 to Aurora A alters substrate and inhibitor interactions. Biochemistry 46, 10 287–10 295 10.1021/bi7011355 (doi:10.1021/bi7011355) 17705509

